# Nonclinical evaluation of HS630, a proposed biosimilar of trastuzumab emtansine: affinity, pharmacokinetics, and immunogenicity

**DOI:** 10.3389/fphar.2025.1698727

**Published:** 2025-12-18

**Authors:** Hui Jiang, Jinjing Che

**Affiliations:** 1 Laboratory of Advanced Biotechnology, Beijing Institute of Pharmacology and Toxicology, Beijing, China; 2 Beijing Institute of Microbiology and Epidemiology, Beijing Institute of Pharmacology and Toxicology, Beijing, China; 3 School of Chemical Engineering, Ocean and Life Sciences, Dalian University of Technology, Panjin, Liaoning, China

**Keywords:** HS630, T-DM1, pharmacokinetics, immunogenicity, affinity

## Abstract

**Objective:**

The aim of this study is to evaluate the similarity of affinity, pharmacokinetics, and immunogenicity shared by HS630 and trastuzumab emtansine (T-DM1).

**Methods:**

*In vitro*, affinity was tested using a Biacore™ T200 apparatus. *In vivo* studies were conducted on tumor-bearing mice and cynomolgus monkeys in the context of different dosages and frequencies of administration. Double-antibody sandwich enzyme-linked immunosorbent assay (ELISA) was used to determine the concentration of total antibody (including naked antibody and antibody–drug conjugate (ADC)) and the ADC of HS630 and Kadcyla®. Furthermore, HPLC–MS/MS was used to determine the concentration of free DM1. The bridge ELISA method was performed to determine anti-drug antibody for immunogenicity analysis.

**Results:**

*In vitro*, HS630 and Kadcyla® exhibited similar binding affinities for human epidermal growth factor receptor 2 (HER2), with K_D_ values of 6.372 E-11M and 9.424 E-11 M, respectively. After injecting 10 mg·kg^−1^ of HS630 and Kadcyla® into tumor-bearing mice, the concentration of total antibody and ADC in serum reached its peak concentration at 5 min, and the concentration of total antibody reached its peak concentration at 24 h in the tumor. Meanwhile, in cynomolgus monkeys, the concentration of total antibody and ADC of HS630 exhibited non-linear pharmacokinetic characteristics following a single intravenous administration of HS630 at 0.33 mg·kg^−1^, 1 mg·kg^−1^, and 3 mg·kg^−1^, respectively. No significant drug accumulation was observed after continuous intravenous administration of 3 mg·kg^−1^ HS630. Biosimilarity evaluation showed that HS630 met the criteria for C_max_ and AUC(0-t) geometric mean ratios with Kadcyla® in the serum of tumor-bearing mice, as well as tumors and serum of cynomolgus monkey. No anti-drug antibody was detected in the serum samples obtained from cynomolgus monkeys after intravenous administration of HS630 and Kadcyla®.

**Conclusion:**

HS630 and originator drug Kadcyla® exhibit pharmacokinetic similarity in tumor-bearing mice and cynomolgus monkeys following intravenous infusion. The comprehensive nonclinical evaluations of this study provide robust evidence for regulatory approval, in addition to addressing of key scientific and technical challenges in biosimilar development.

## Graphical Abstract

**Figure:**
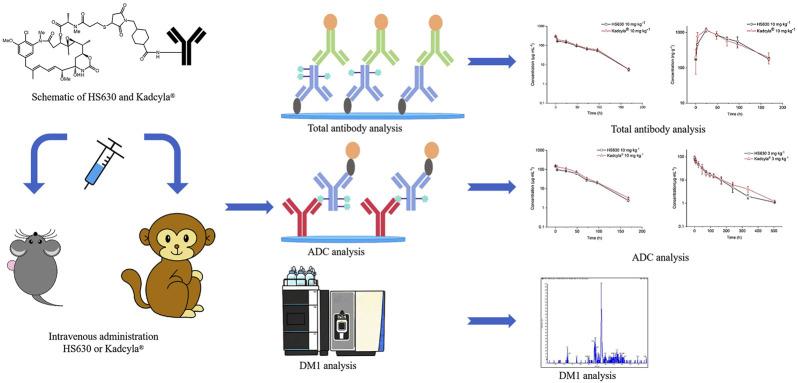

## 1 Introduction

There is currently a remarkable surge in the incidence of malignant tumors in China with the increase in environmental pollution and life stress, accompanied by a distinctly younger onset (Zheng et al., 2022; Han et al., 2024). However, conventional chemotherapeutic drugs have poor targeting and strong toxic side effects, highlighting an urgent need to develop more targeted antitumor drugs (Juthani et al., 2024). The ideal drug should integrate both target-specific antibodies and small molecule drugs of excellent clinical efficacy—antibody–drug conjugate (ADC). The structure of ADC formulation consists of a monoclonal antibody (mAb) with a targeting effect linked to a compound with specific pharmacological properties, such as cytotoxic effects (Udofa et al., 2024). As of July 2025, 19 ADC drugs have been approved worldwide. However, in actual practice, it is a challenge to target the chemotherapeutic agent to tumor cells using an mAb. The mAb must be conjugated to a cytotoxin with therapeutic effect, and this binding must be sufficiently stable to ensure minimal release of the cytotoxic payload into the systemic circulation. In the case of destabilized binding, cytotoxin would be released and ultimately internalized by the target cells (Wang et al., 2023; Kumari et al., 2024). Moreover, the process of coupling a cytotoxin to an mAb may produce an ADC, which should maintain target recognition of the target after coupling (Colombo et al., 2024; Li et al., 2025). Simultaneously, in order to minimize batch-to-batch variability, it is critical to strictly control the amount of cytotoxin bound to each intact mAb, such as the drug-to-antibody ratio (DAR) and the level of unbound antibody. It is exceptionally complex to evaluate batch differences given that each of the three main components of ADC must meet stringent criteria (Ji et al., 2015; Bechtold-Peters et al., 2023; Li et al., 2024).

**FIGURE 1:**
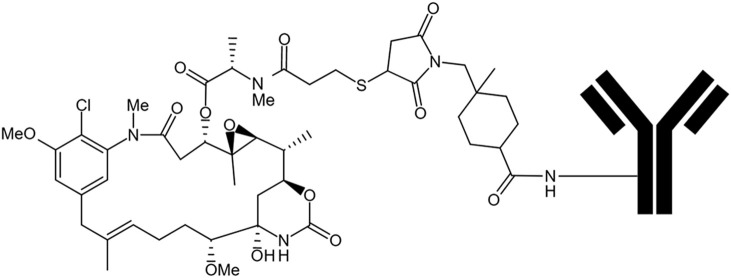
Schematic of trastuzumab emtansine (T-DM1).

Trastuzumab emtansine (T-DM1) is an ADC developed by Roche, which consists of humanized mAb trastuzumab (also known as Herceptin) and a cytotoxic drug called maytansine derivative (DM1). It was approved by the US Food and Drug Administration (FDA) in 2013 for the treatment of advanced human epidermal growth factor receptor 2 (HER2, as known as ErbB2)-positive breast cancer. The schematic of T-DM1 is shown in Figure 1 (Lewis Phillips et al., 2008; Krop et al., 2010; von Arx et al., 2023). Trastuzumab can specifically target HER2 in breast and gastric cancer, while emtansine is a synthetic derivative of maytansine (a small molecule cytotoxin that binds to tubulin, preventing microtubule formation through a non-reduced bis-maleimide monopropylenediol conjugate). Trastuzumab was approved only for HER2-positive cancers and is not capable of precipitating apoptosis in all HER2-positive cells. In response to this issue, trastuzumab and emtansine were integrated into an ADC, resulting in a proportional binding of the antibody to the HER2 receptor that can induce cellular internalization of maytansine released from the conjugate, leading to the killing of tumor cells (Lambert and Chari, 2014). Both *in vitro* and *in vivo* studies revealed that, compared with trastuzumab, T-DM1 has better overall efficacy and pharmacokinetic properties and less toxicity. Clinical trials also suggested that the survival rate of patients treated with T-DM1 is higher than those treated with trastuzumab alone (Minckwitz et al., 2019; Geyer et al., 2025). Critically, HS630 is a proposed T-DM1 biosimilar under development by Zhejiang Hisun pharmaceutical industry.

A biosimilar is a therapeutic biological product with demonstrably similar quality, safety, and efficacy to an approved originator drug (Schellekens et al., 2016; Perez et al., 2017). While regulatory agencies such as the FDA, National Medical Products Administration (NMPA), and World Health Organization (WHO) may vary in their descriptions of the similarity of biosimilars, the core concept of biosimilar similarity is a comprehensive assessment based on evidence chain from pharmaceutical, nonclinical, and clinical trials (Kurki et al., 2022; Kang et al., 2023). Quality similarity studies are integral to the entire development of biosimilars. They aim to ensure the consistency of the proposed biosimilar with the originator product in the structure, physical and chemical properties, purity and impurities, biological functions, immunological characteristics, biological activity, pharmacokinetics, pharmacodynamics, efficacy, safety, immunogenicity, and other attributes, as well as no clinical differences (Verrill et al., 2019; Mascarenhas-Melo et al., 2024; Monga et al., 2025).

Accordingly, this study was conducted to assess the similarity between HS630 and the original drug with respect to *in vitro* affinity, nonclinical pharmacokinetics, immunogenicity, and distribution in target tissues.

## 2 Materials and methods

### 2.1 Reagents

The study drug: HS630 (recombinant anti-HER2 humanized mAb conjugated to maytansine derivative DM1 for injection), obtained from Zhejiang Hisun Pharmaceutical Co., Ltd., lot no.: 20131101, specification: 100 mg, sterile powder for injection, stored at 5 °C ± 3 °C. Control drug: Kadcyla® (Trastuzumab emtansine, T-DM1), manufactured by Roche and provided by Zhejiang Hisun Pharmaceutical Co., Ltd., lot no.: 566313, specification: 100 mg/vial, sterile powder for injection, stored at 5 °C ± 3 °C. DM1 standard, lot no.: S140101, white powder, 99.3% purity, Zhejiang Hisun Pharmaceutical Co., Ltd. Internal standard: Maytansinol, lot no.: S131102, white powder, >93% purity, Zhejiang Hisun Pharmaceutical Co., Ltd. All were stored at −20 °C ± 5 °C in the dark. Anti-DM1 antibody: Zhejiang Hisun Pharmaceutical Co., Ltd., concentration: 4 mg·mL^−1^, stored at −80 °C. Recombinant human ErbB2 (also known as HER2): Sino Biological Inc., catalog: 100040-H08H, lot no.: LC08SE2313, stored at −20 °C. Biotin Labeling Kit-NH2: Dojindo Molecular Technologies. Inc. Corp., lot no.: FN744, goat anti-human IgG-heavy and light chain monkey-adsorbed antibody HRP-conjugated: Bethyl Laboratories, Inc., catalog: A80-319P, lot no.: A80-319P-15, stored at 2 °C–8 °C. TMB single component color development solution: Beijing Solai Bao Technology Co., Ltd., catalog: PR1200; lot no.: 20140625.

### 2.2 Animals

This study involved thirty cynomolgus monkeys (laboratory animal qualifying number 45001200000032), obtained from Guangxi Guidong Primate Development Experiment Co., Ltd. (experimental animal production license number SCXK (GUI) 2011-0001) (both sexes, weighing 3.2 ± 0.4 kg). All experimental monkeys were fed in separate cages with free access to water and fresh fruit twice daily in good feeding condition. Female BALB/cA-nude mice (5–6 weeks old, SPF-grade) were purchased from Shanghai Lingchang Biotech Co. Ltd. (experimental animal production license number: SCXK (Hu)2013-0018; laboratory animal qualifying number: 2013001801695; weighing 15–20 g). Animal welfare and experimental procedures and related ethical regulations were carried out strictly in accordance with the *Guide for the Care and Use of Laboratory Animals*.

### 2.3 Affinity studies *in vitro*

The experiments were conducted using a Biacore™ T200 apparatus (GE Healthcare Life Sciences, Uppsala, Sweden) on Sensor Chip CM5 (Biacore™) at 25 °C. The active and reference surfaces were the flow cell (Fc2) bound to HS630 and Kadcyla® and the flow cell (Fc2) not bound to HS630 and Kadcyla®, respectively. HBS-EP + buffer solution was used as the mobile phase to achieve similar response values for the next test, with 3 M MgCl_2_ as the regeneration solution. After testing, all samples were finally bound to the active surfaces at 333 ng·mL^−1^. Then, different concentrations of HER2 solutions, diluted by a factor of running buffer based on the surface test, to formulate standards with HER2 concentration gradients of 10, 5, 2.5, 1.25, 0.625, and 0 nM at different concentration, were injected through both active and reference surfaces. Finally, the sample were injected at a flow rate of 30 µL·min^−1^ with 3 min association time and 30 min dissociation time.

### 2.4 Precision and accuracy verification by bioanalysis

ADC and total antibody concentrations in serum samples were quantified using methodologically validated enzyme-linked immunosorbent assay (ELISA). The small molecule fraction was detected using an LC–MS/MS method. Immunogenicity analysis was performed by the bridge ELISA method. According to the methodological reliability test, the established HPLC–MS/MS for the DM1 concentration determination in monkey and mice serum exhibited high specificity, sensitivity, precision, and stability, meeting the requirements of pharmacokinetic study.

### 2.5 Pharmacokinetics and tissue distribution in tumor-bearing mice

Following subcutaneous inoculation of BT-474/T721 cells, BALB/cA-nude mice were randomly divided into groups when the tumor volume reached 300–400 mm^3^. The experiment design is shown in Table 1. There were three single intravenous administration HS630 groups (10 mg·kg^−1^) and three single intravenous administration Kadcyla® groups (10 mg·kg^−1^). Serum and tumor samples of mice were collected at 0.083 h, 4 h, 24 h, 48 h, 72 h, 96 h, and 168 h post-dose, respectively.

**TABLE 1:** Animal grouping and sample collection design.

Group	Dose	Animal no. for serum collection						
		5 min	4 h	24 h	48 h	72 h	96 h	168 h
HS630	10 mg·kg^−1^	1# 2# 3#	4# 5# 6#	7# 8# 9#	10# 11# 12#	13# 14# 15#	16# 17# 18#	19# 20# 21#
Kadcyla®		22# 23# 24#	25# 26# 27#	28# 29# 30#	31# 32# 33#	34# 35# 36#	37# 38# 39#	40# 41# 42#
Control	-	43#44#45#46#						

### 2.6 *In vivo* pharmacokinetics and immunogenicity in cynomolgus monkeys

**FIGURE 2:**
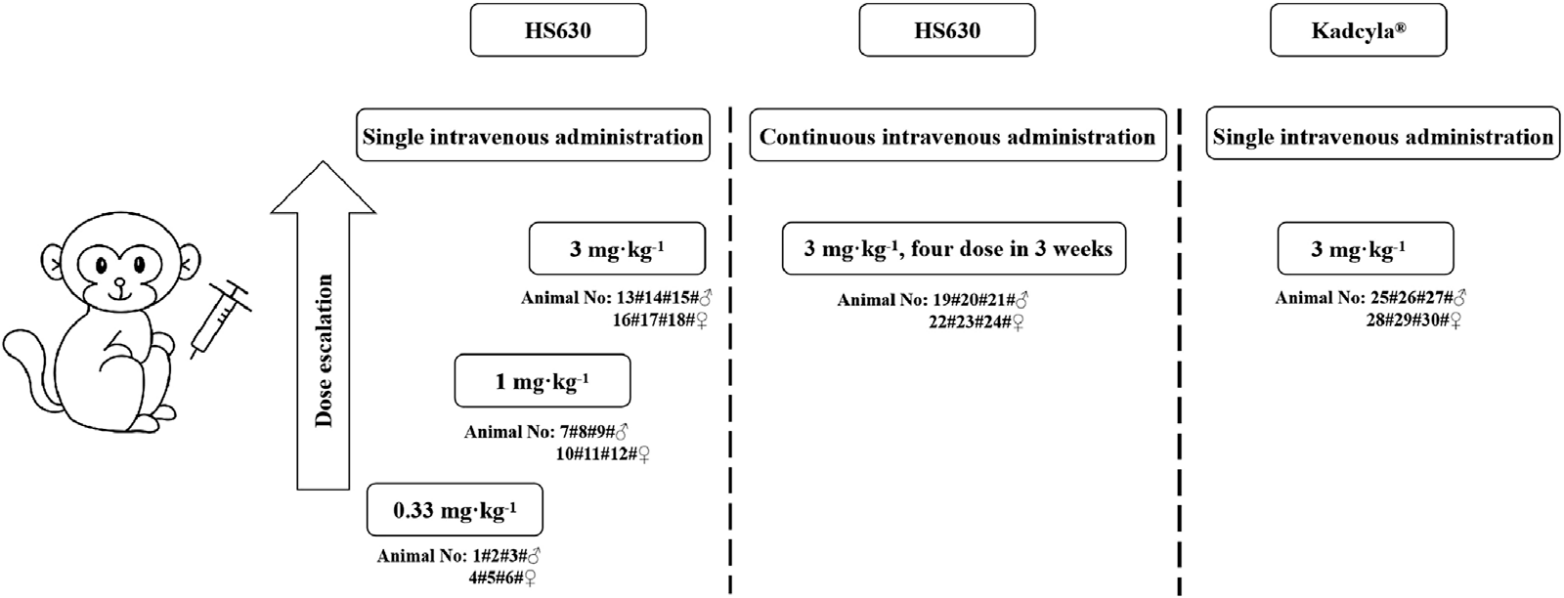
Dose design and grouping.

In this experiment, six cynomolgus monkeys (half male and half female) were assigned to each dose group, with corresponding animal numbers shown in Figure 2. This involved three single intravenous administration HS630 groups (0.33, 1, and 3 mg·kg^−1^), one continuous intravenous administration HS630 group (3 mg·kg^−1^), and one single intravenous administration control Kadcyla® group (3 mg·kg^−1^). For the single intravenous administration groups of HS630 and the control Kadcyla® group, cynomolgus monkey serum was collected at 0 h pre-dose and 0.5 h, 4 h, 8 h, 24 h, 48 h, 72 h, 96 h, 120 h, 168 h, 240 h, 336 h, and 504 h post-dose. For the continuous intravenous administration HS630 group, serum was collected from cynomolgus monkeys 0 h before drug administration, 0.5 h after the first dose, 4 h, 8 h, 24 h, 48 h, 72 h, 96 h, 120 h, 168 h, and 336 h before the second dose, 0.5 h after the second dose and before the third dose, 0.5 h after the third dose and before the fourth dose, and 0.5 h, 4 h, 8 h, 24 h, 48 h, 72 h, 96 h, 120 h, 168 h, 336 h, 504 h, 672 h, 840 h, and 1,008 h after the fourth dose.

### 2.7 Establishment and validation of the analytical method

#### 2.7.1 Establishment and validation of the ELISA method for ADC

In this method, the ADC was quantified by double-antibody sandwich ELISA. The anti-DM1 mAb was coated on a 96-well plate as the primary antibody, with the addition of a series of concentrations of standard solution or diluted serum samples. The ADC in the standard solution or serum would specifically bind to the primary antibody, followed by supplementation of the diluted goat anti-human IgG-HRP (secondary antibody) adsorbed by monkey serum. The secondary antibody specifically bound to the ADC, which bound to the primary antibody. The next step was the addition of the substrate chromogenic agent. Finally, it was terminated with stop solution sulfuric acid, after which optical density (OD) values were detected using a microplate reader within a wavelength range of 450 nm–560 nm. The depth of color was proportional to the concentration of the ADC in the sample.

#### 2.7.2 Establishment and validation of the ELISA method for total antibody

In this method, total antibody was quantified using the double-antibody sandwich ELISA. Recombinant human HER2 was used as the primary antibody to coat the 96-well plate, followed by the addition of a series concentration of standard solution or diluted serum samples. The total antibody in the standard solution or serum would specifically bind to the primary antibody, after which the diluted goat anti-human IgG-HRP (secondary antibody) adsorbed by monkey serum was added. The secondary antibody specifically bound to the total antibody, which bound to the primary antibody, with the addition of the substrate chromogenic agent subsequently. Finally, it was terminated with stop solution sulfuric acid, and the OD values were detected using a microplate reader within 450 nm–560 nm. The depth of color was proportional to the concentration of total antibody in the sample.

#### 2.7.3 Validation of HPLC–MS/MS for detecting DM1

HPLC–MS/MS was used to determine the concentration of free DM1 in the serum of cynomolgus monkeys, combined with an investigation of the distribution characteristics of DM1 in cynomolgus monkey after intravenous administration of HS630 and Kadcyla® at different doses for injection. Consequently, the specificity, sensitivity, precision, and stability of the established HPLC–MS/MS met the requirements for pharmacokinetic studies.

### 2.8 Establishment of the ELISA for the anti-HS630 antibody

#### 2.8.1 DM1-positive determination by screening bridge ELISA

Based on the cut-off value, samples were determined to be negative (below cut-off) or positive (above cut-off). A bridge ELISA assay was performed on the serum samples of 60 healthy normal cynomolgus monkeys, which were assayed three times in parallel. The cut-off value was calculated as the average of serum samples from each batch of negative samples plus thrice the standard deviation. Finally, the average of the three batches of critical values for this test was calculated to determine the negative or positive result.

#### 2.8.2 ELISA

The concentration of anti-HS630 antibody in serum was investigated by the ELISA. The test sample and negative controls were added after coating a 96-well plate with the HS630. Then, the HS630 was detected by biotin labelled, followed by signal amplification through the addition of Hrp–strep, with the recording of the absorbance values at 450 nm–560 nm. Samples with OD above the cut-off value were considered positive.

#### 2.8.3 Corroboration: immune clearance

The serum samples, which were determined to be positive by the bridge ELISA, were further confirmed for the presence of monkey anti-HS630 antibody by immune clearance.

1. Principle of the assay. Excess HS630 was added in serum samples which tested positive. The excess HS630 would compete with the coated HS630 for binding to monkey anti-HS630 antibody when the sample contained monkey anti-HS630 antibody. The bound antibody would be removed from the liquid phase of this fraction during the wash process, ultimately resulting in a reduced signal value.
2. Test methods. Serum samples from each time point of collecting serum samples that tested positive by the screening method were subjected to an immune clearance test. The samples contained anti-HS630 antibody and were judged to be positive when the signal values were significantly reduced in the control group compared to the group without and with the addition of HS630 (*p* < 0.05). False positives were judged if there was no significant difference in the signal values (*p* > 0.05) and if the positive result was likely due to non-specific binding of the serum matrix.

## 3 Results

### 3.1 Affinity studies *in vitro*

**FIGURE 3:**
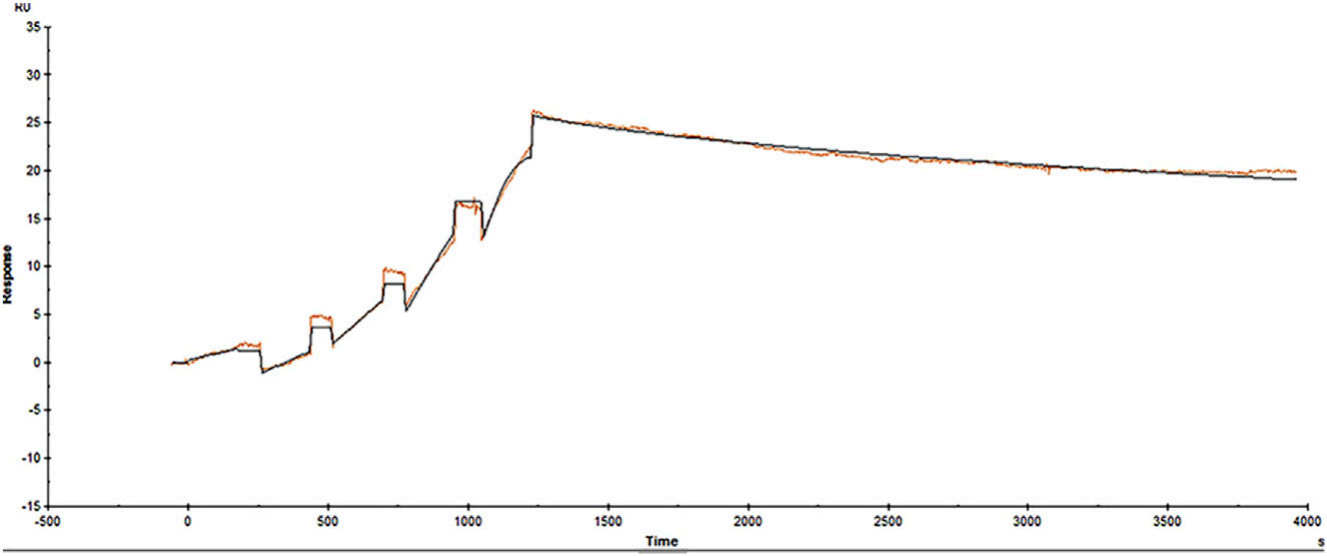
Binding kinetic curves of HS630 to HER2.

**FIGURE 4:**
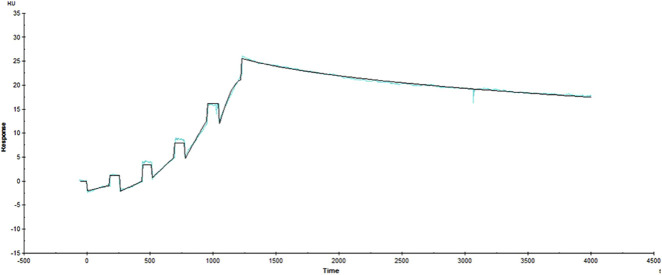
Binding kinetic curves of Kadcyla® to HER2.

Surface plasmon resonance (SPR) signals were acquired and saved using Biacore™ T200 control software. Each concentration of a subject injected onto a flow cell (FC2) incorporating HS630 and Kadcyla® was corrected by subtracting the corresponding reference flow cell (FC1) profile. Simultaneously, a 1:1 binding kinetics model was used for curve fitting to most appropriately describe the reaction kinetics and calculate the K_a_, K_d_, and K_D_ values. The binding signal response value at the end of the experiment was similar to that at its beginning. Therefore, the receptor binding was stable and multiple regenerations produced no impact on the binding ability to the ligand, supporting its repeated utilization after activation. Figures 3, 4 show the fitted curves (black lines) for the binding kinetics curves (colored lines) of HER2 with HS630 and Kadcyla® after treatment with 1:1 binding model, respectively. The kinetic parameters obtained are listed in Table 2. The K_D_ values of HS630 and Kadcyla® for HER2 were 6.372 E-11M and 9.424 E-11 M, respectively.

**TABLE 2:** Affinity constants of HER2 with HS630 and Kadcyla^®^.

	ka (M^-1^s^-1^)	kd (s^-1^)	K_D_ (M)
HS630	3.777 E+6	2.407 E-4	6.372E-11
Kadcyla®	3.705 E+6	3.491 E-4	9.424E-11

### 3.2 Bioanalysis

The linear range of HS630 ELISA was 1.5625–100 ng·mL^−1^, and the recovery of LOQ (1.5625 ng·mL^−1^) was evaluated as intra-assay CV% <20% (4.47%). Intra-assay precision was 7.77%, 5.74%, and 7.16%, and intra-assay accuracy was +1.08%, +3.67%, and +2.13% for the high, medium, and low concentrations, respectively. Inter-assay precision was 8.06%, 5.40%, and 8.68%, and inter-assay accuracy was −3.61%, +1.66%, and −5.88% for the high, medium, and low concentrations, respectively. This method had excellent specificity without cross-reactivity of trastuzumab, bevacizumab, adalimumab, or infliximab. HS630 revealed no altered stability after storage in serum at −80 °C (after three freeze/thaw cycles), room temperature for 4 h, 4 °C for 4 h, and −80 °C for 40 and 100 days, but its stability could not be ensured following long-term storage at −80 °C for 160 days. Therefore, sample testing was completed before 160 days of storage. In addition, 12,500-, 62,500-, and 500,000-fold dilutions also produced no effect on sample detection. The methodological validation showed that the specificity, precision, and accuracy of the ELISA method for measuring HS630 concentrations in monkey serum fulfilled the requirements of pharmacokinetic studies. Supplementary Appendixes 1–3 summarize the method validation of ELISA and HPLC–MS/MS.

### 3.3 Pharmacokinetics and tissue distribution in tumor-bearing mice

**FIGURE 5:**
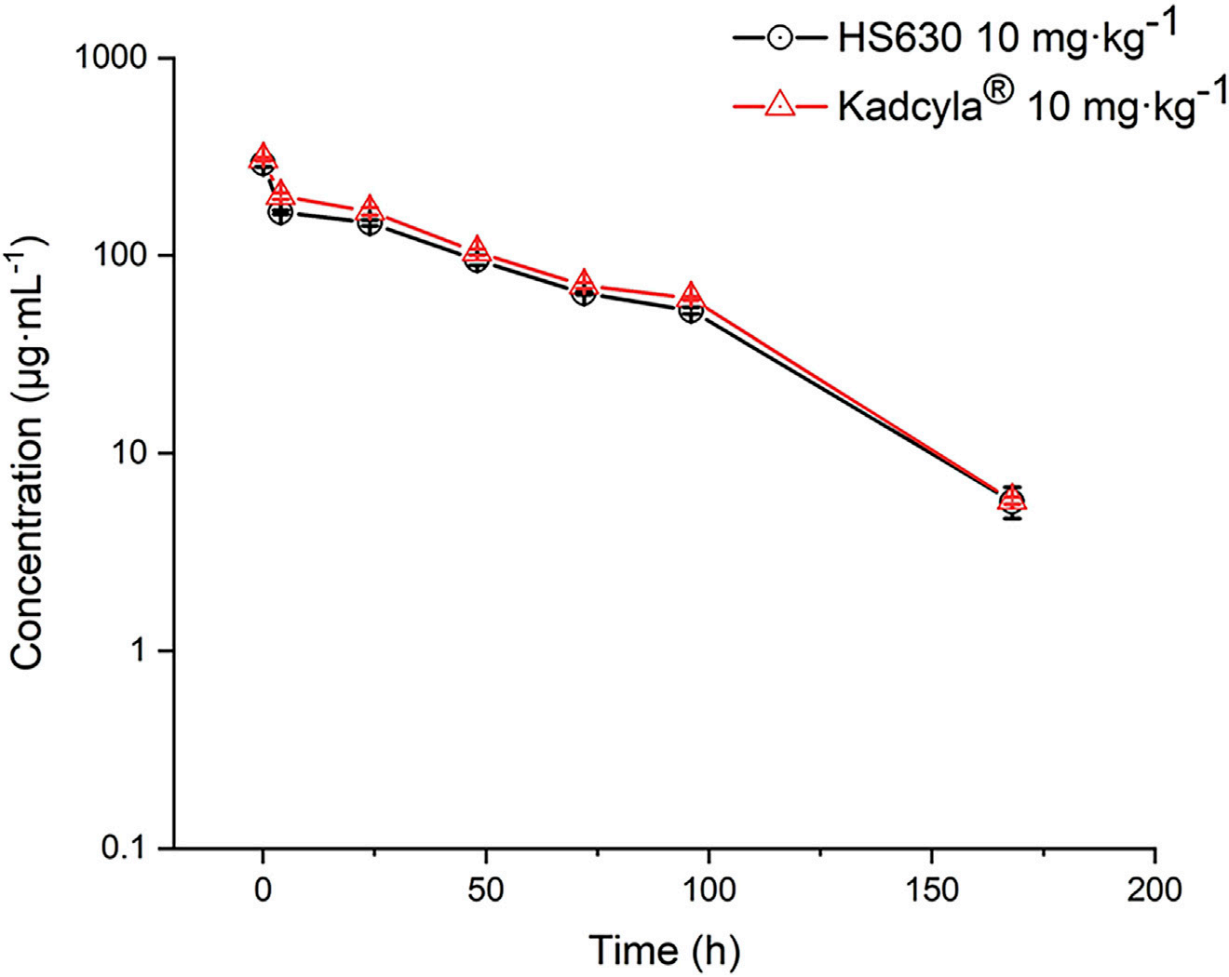
Comparison of total antibody (including naked antibody and ADC) concentration in serum after tail vein injection of 10 mg·kg^−1^ HS630 and Kadcyla® in tumor-bearing mice (n = 3).

**FIGURE 6:**
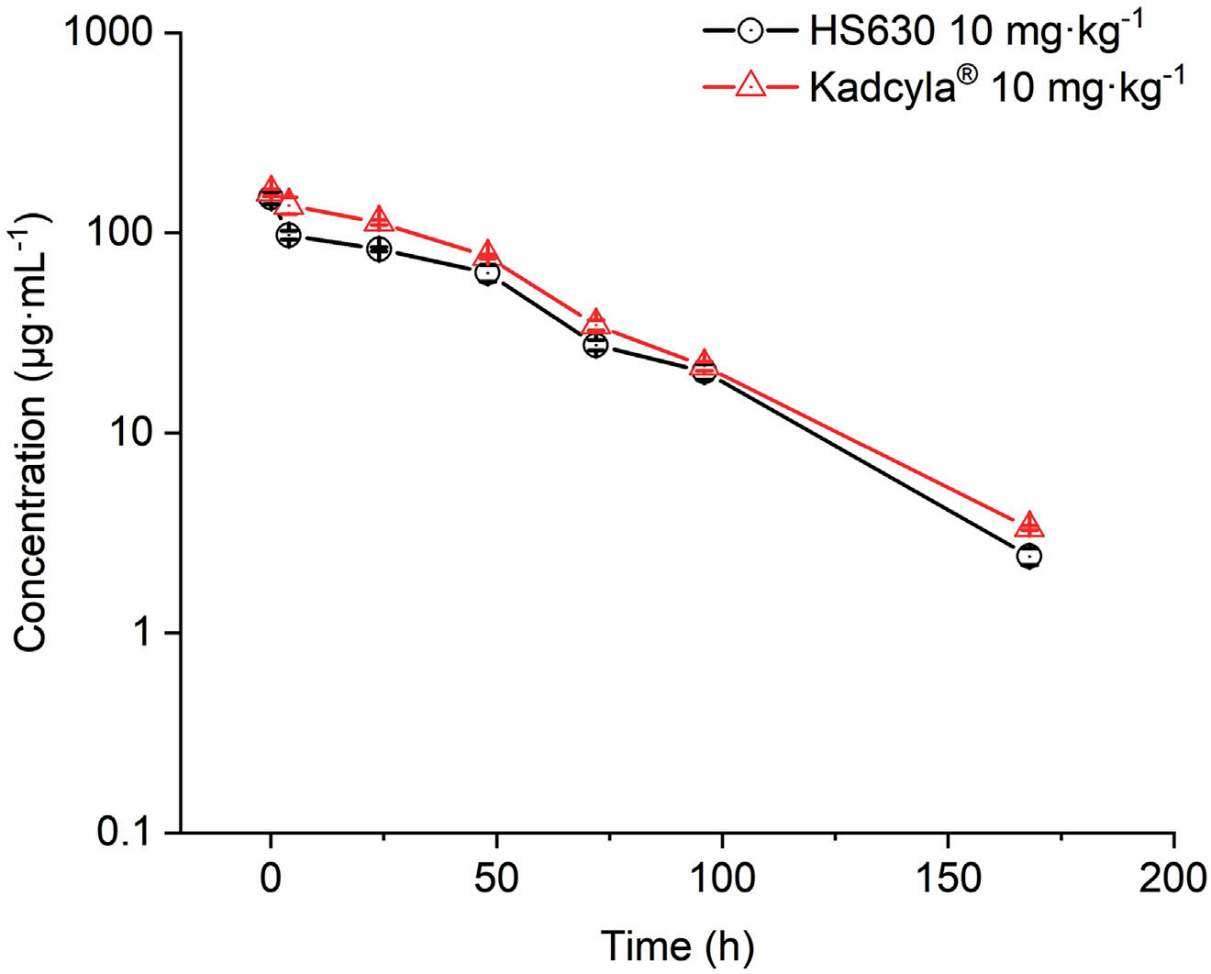
Comparison of ADC in the serum of tumor-bearing mice after tail vein injection of 10 mg·kg^−1^ HS630 and Kadcyla® (n = 3).

In this experiment, 10 mg·kg^−1^ HS630 and Kadcyla® were injected via the tail vein into tumor-bearing mice. Consequently, in serum, the peak concentration C_max_ of total antibody was 292.038 μg·mL^−1^ and 307.519 μg·mL^−1^, respectively; time to peak concentration T_max_ was 0.083 h; AUC_(0–t)_ was 12288.120 h·μg·mL^−1^ and 13934.246 h·μg·mL^−1^, respectively; AUC_(0–inf)_ was 12543.592 h·μg·mL^−1^ and 14185.157 h·μg·mL^−1^, respectively; apparent distribution volume (V_SS_) was 37.549 mL·kg^−1^ and 33.005 mL·kg^−1^, respectively; clearance (CL) was 0.797 mL·h^−1^·kg^−1^ and 0.705 mL·h^−1^·kg^−1^, respectively; terminal elimination half-life (T_1/2_) was 31.076 h and 30.130 h, respectively; and mean residence time (MRT) was 47.113 h and 46.816 h, respectively. WinNonlin was used for biosimilarity evaluation. The serum concentration–time curve is shown in Figure 5, and the pharmacokinetics parameters are shown in Table 3. HS630 showed the same trend as Kadcyla®. Simultaneously, ADC C_max_ was 149.962 μg·mL^−1^ and 159.041 μg·mL^−1^; T_max_ was 0.083 h; AUC_(0–t)_ was 6,517.445 h·μg·mL^−1^ and 8,129.677 h·μg·mL^−1^; AUC_(0–inf)_ was 16613.594 μg·mL^−1^ and 8,264.784 h·μg·mL^−1^; V_SS_ was 63.330 mL·kg^−1^ and 49.800 mL·kg^−1^; CL was 1.512 mL·h^−1^·kg^−1^ and 1.210 mL·h^−1^·kg^−1^; T_1/2_ was 27.610 h and 27.944 h; and MRT was 41.885 h and 41.157 h, respectively. The serum concentration–time curve is presented in Figure 6, and the pharmacokinetics parameters are listed in Table 4. HS630 showed the same trend as Kadcyla®.

**TABLE 3:** Comparison of total antibody (including naked antibody and ADC) parameters in serum of tumor-bearing mice after tail vein injection of 10 mg·kg^−1^ HS630 and Kadcyla^®^.

Parameter	HS630	Kadcyla®
AUC_(0–t)_ (µg·h·mL^−1^)	12288.120	13934.246
AUC_(0–inf)_ (µg·h·mL^−1^)	12543.592	14185.157
MRT(h)	47.113	46.816
CL (mL·kg^−1^·h^−1^)	0.797	0.705
V_ss_ (mL·kg^−1^)	37.549	33.005
T_1/2_ (h)	31.076	30.130
kel	0.022	0.023
C_max_ (µg·mL^−1^)	292.038	307.519
T_max_ (h)	0.083	0.083
C_max_ geometric mean ratio	89.205%	
AUC_(0–t)_ geometric mean ratio	88.186%	

**TABLE 4:** Comparison of ADC parameters in the serum of tumor-bearing mice after tail vein injection of 10 mg·kg^−1^ HS630 and Kadcyla^®^.

Parameter	HS630	Kadcyla®
AUC_(0-–t)_ (µg·h·mL^−1^)	6,517.445	8,129.677
AUC_(0-–inf)_ (µg·h·mL^−1^)	6,613.594	8,264.784
MRT (h)	41.885	41.157
CL (mL·kg^−1^·h^−1^)	1.512	1.210
V_ss_ (mL·kg^−1^)	63.330	49.800
T_1/2_ (h)	27.610	27.944
kel	0.025	0.025
C_max_ (µg·mL^−1^)	149.962	159.041
T_max_ (h)	0.083	0.083
Cmax geometric mean ratio	89.270%	
AUC_(0–t)_ geometric mean ratio	80.169%	

**FIGURE 7:**
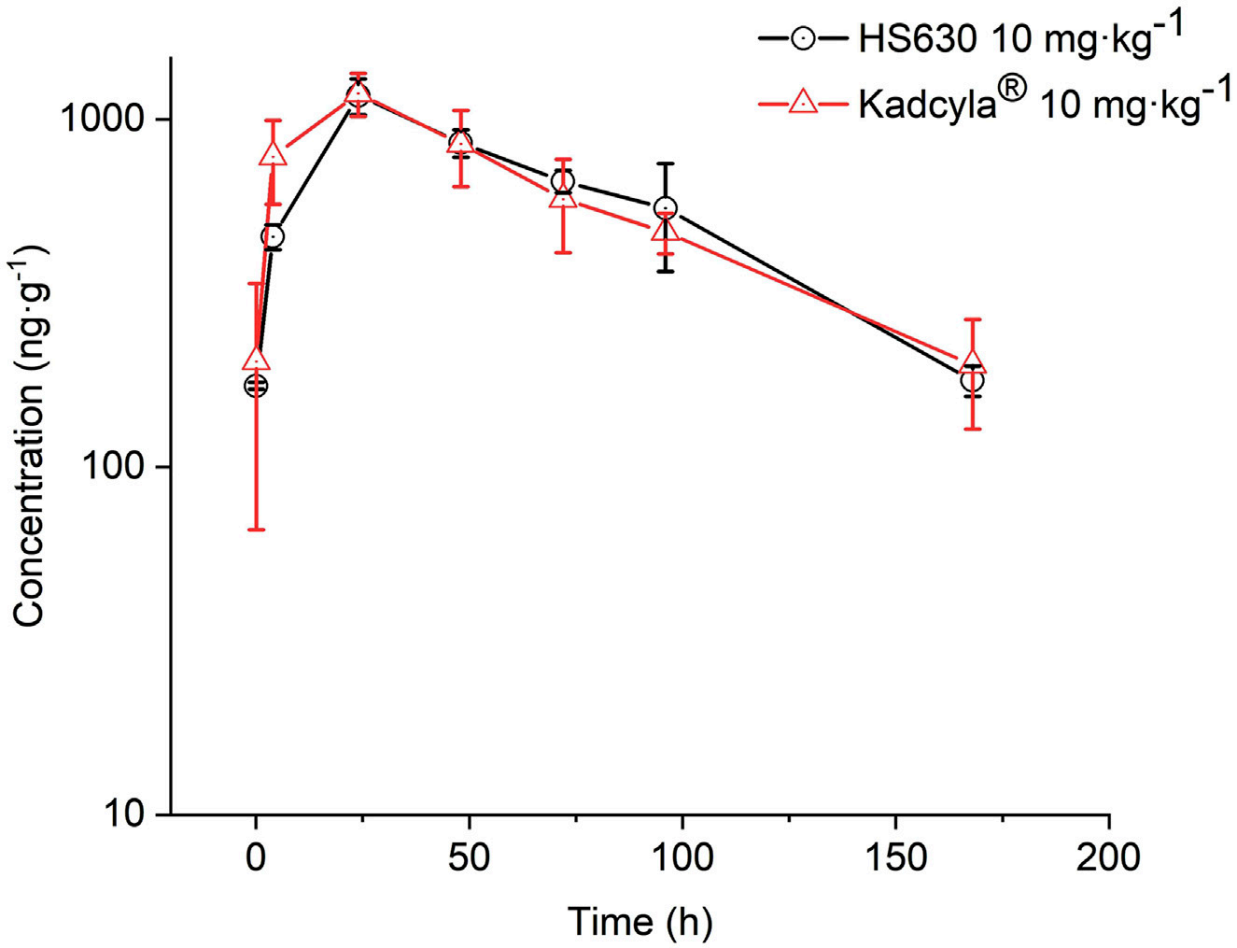
Comparison of total antibody (including naked antibody and ADC) concentration in tumor after tail vein injection of 10 mg·kg^−1^ HS630 and Kadcyla® in tumor-bearing mice (n = 3).

After the tail vein injection of 10 mg·kg^−1^ HS630 and Kadcyla® in tumor-bearing mice, in the tumors, C_max_ was 1.170 μg·g^-1^ and 1.187 μg·g^-1^; AUC_(0–t)_ was 98.692 h·μg·g^-1^ and 97.536 h·μg·g^-1^; AUC_(0–inf)_ was 112.438 h·μg·g^-1^ and 113.429 h·μg·g^-1^; V_SS_ was 5,471.822 g·kg^−1^ and 5,569.924 g·kg^−1^; CL was 88.938 g·h^−1^·kg^−1^ and 88.161 g·h^−1^·kg^−1^; T_1/2_ was 53.721 h and 55.887 h; and MRT was 61.524 h and 63.179 h, respectively. The tumor concentration–time curve is depicted in Figure 7, and the parameters of total antibody concentration of HS630 and Kadcyla® in the tumors are provided in Table 5. The trend of HS630 was consistent with that of Kadcyla®.

**TABLE 5:** Comparison of total antibody (including naked antibodies and ADC) parameters in tumor after tail vein injection of 10 mg·kg^−1^ HS630 and Kadcyla^®^ in tumor-bearing mice.

Parameter	HS630	Kadcyla®
AUC_(0-–t)_ (µg·h·g^−1^)	98.692	97.536
AUC_(0-–inf)_ (µg·h·g^−1^)	112.438	113.429
MRT (h)	61.524	63.179
CL (g·kg^−1^·h^−1^)	88.938	88.161
V_ss_ (g·kg^−1^)	5,471.822	5,569.924
t_1/2_ (h)	53.721	55.887
kel	0.013	0.012
C_max_ (µg·g^−1^)	1.170	1.187
T_max_ (h)	24	24
C_max_ geometric mean ratio	128.036%	
AUC_(0-–t)_ geometric mean ratio	101.185%	

In addition, irregular ADC data in tumors of tumor-bearing mice at each time point after tail vein injection of 10 mg·kg^−1^ HS630 and Kadcyla® were observed. Owing to the detection method, there was no trend on peak increasing or decreasing. The anti-DM1 antibody was used as the coating primary antibody to detect ADC, which was connected with the DM1 part of ADC, while goat anti-human IgG-HRP adsorbed by HRP-labeled monkey serum was used as the secondary antibody. The secondary antibody for detection was connected with the macromolecular antibody part of the ADC, but the ADC cannot be detected, given that the link bond between the macromolecule and DM1 was broken in the tumors. The specific data of each sample is summarized in Supplementary Appendix 4.

### 3.4 *In vivo* pharmacokinetics in cynomolgus monkeys

#### 3.4.1 Pharmacokinetic parameters of the ADC after a single intravenous infusion of HS630 at different doses in cynomolgus monkeys

**FIGURE 8:**
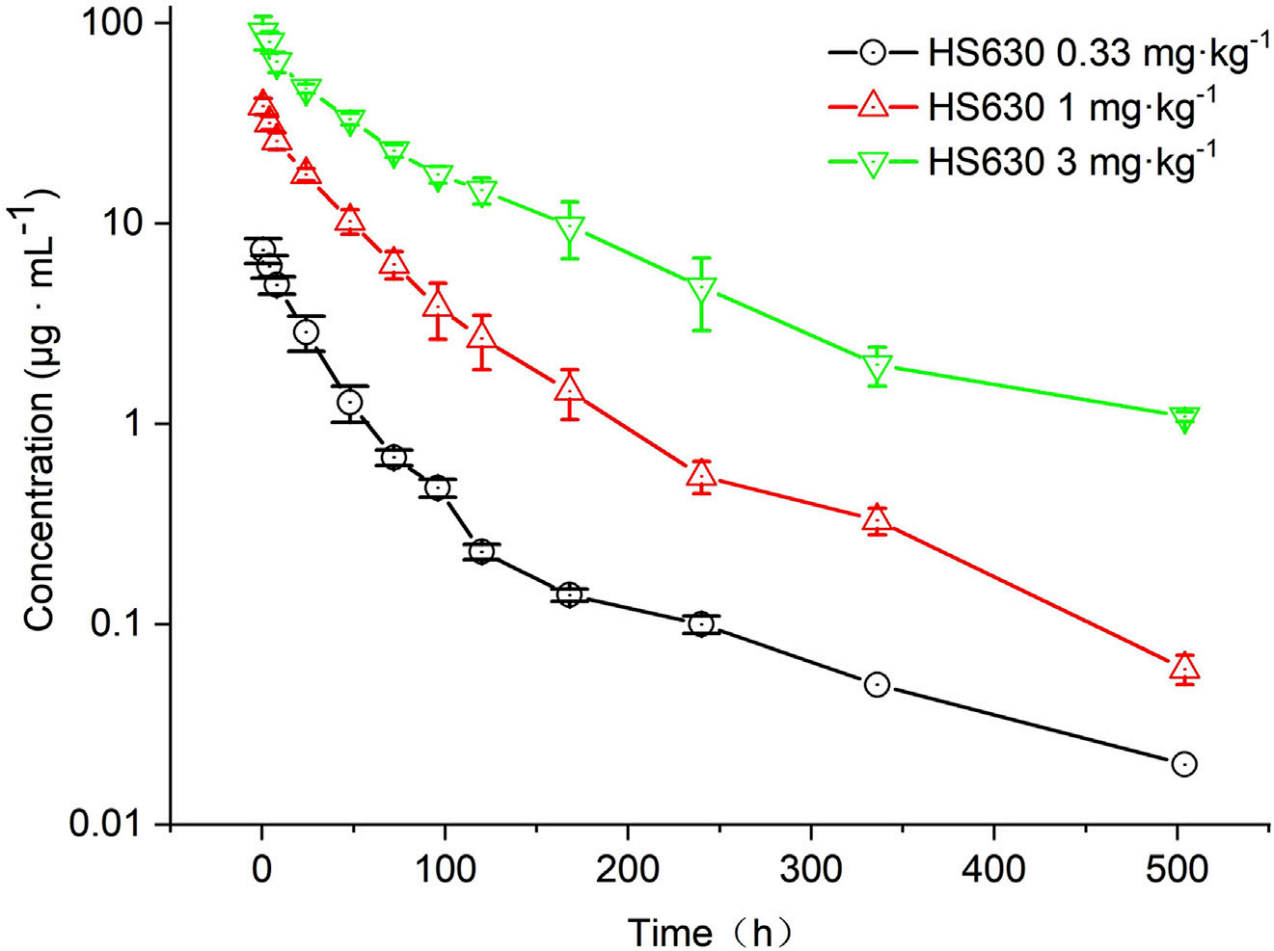
ADC serum concentration–time curves of HS630 after single intravenous infusion at different doses in cynomolgus monkeys.

Figure 8 shows the concentration of ADC in the serum of cynomolgus monkeys after a single intravenous infusion of 0.33 mg·kg^−1^, 1 mg·kg^−1^, and 3 mg·kg^−1^ HS630; Table 6 describes the ADC pharmacokinetic parameters of HS630 at these doses. The post-administration T_1/2_ were 121.81 ± 4.71 h, 74.05 ± 5.63 h, and 101.68 ± 8.05 h, respectively, while AUC_(0–504 h)_ were 235.74 ± 23.47 μg·h·mL^−1^, 1,573.49 ± 139.77 μg·h·mL^−1^ and 5,712.66 ± 528.5 μg·h·mL^−1^, respectively, showing statistically significant differences among the groups (*p* < 0.01). CL was 1.39 ± 0.13 mL·kg^−1^·h^−1^, 0.64 ± 0.06 mL·kg^−1^·h^−1^, and 0.51 ± 0.05 mL·kg^−1^·h^−1^, respectively. T_max_ was all at 0.5 h after administration, and C_max_ was 7.37 ± 1.04 μg·mL^−1^, 38.56 ± 3.61 μg·mL^−1^, and 93.56 ± 14.21 μg·mL^−1^, respectively. The dose ratio was 1:3:9, the ratio of AUC_(0–504 h)_ was 1:6.67: 24.23, and the ratio of C_max_ was 1:5.23: 12.69. Therefore, HS630 exhibited non-linear pharmacokinetic characteristics in cynomolgus monkeys, within a dose range of 0.33∼3 mg·kg^−1^.

**TABLE 6:** Pharmacokinetic parameters of ADC after single intravenous infusion at different doses of HS630 in cynomolgus monkeys.

Parameter	Unit	Low dose (0.33 mg·kg^−1^)	Medium dose (1 mg·kg^−1^)	High dose (3 mg·kg^−1^)
AUC_(0–504 h)_	μg·h·mL^−1^	235.74 ± 23.47	1,573.49 ± 139.77	5,712.66 ± 528.5
AUC_(0–inf)_	μg·h·mL^−1^	240.05 ± 23.35	1,580.17 ± 140.08	5,872.89 ± 521.13
AUC_(504 h–inf)_	μg·h·mL^−1^	4.31 ± 0.37	6.69 ± 1.45	160.23 ± 9.74
MRT	h	55.92 ± 3.7	61.56 ± 1.76	94.08 ± 8.45
CL	mL·kg^−1^·h^−1^	1.39 ± 0.13	0.64 ± 0.06	0.51 ± 0.05
V_ss_	mL·kg^−1^	77.85 ± 12.21	39.24 ± 3.95	48.08 ± 1.35
T_1/2_	h	121.81 ± 4.71	74.05 ± 5.63	101.68 ± 8.05
Kel	h^−1^	0.01 ± 0	0.01 ± 0	0.01 ± 0
C_max_	μg·mL^−1^	7.37 ± 1.04	38.56 ± 3.61	93.56 ± 14.21
T_max_	h	0.5 ± 0	0.5 ± 0	0.5 ± 0

#### 3.4.2 Pharmacokinetic parameters of the ADC after continuous intravenous administrations of high-dose HS630 in cynomolgus monkeys

**FIGURE 9:**
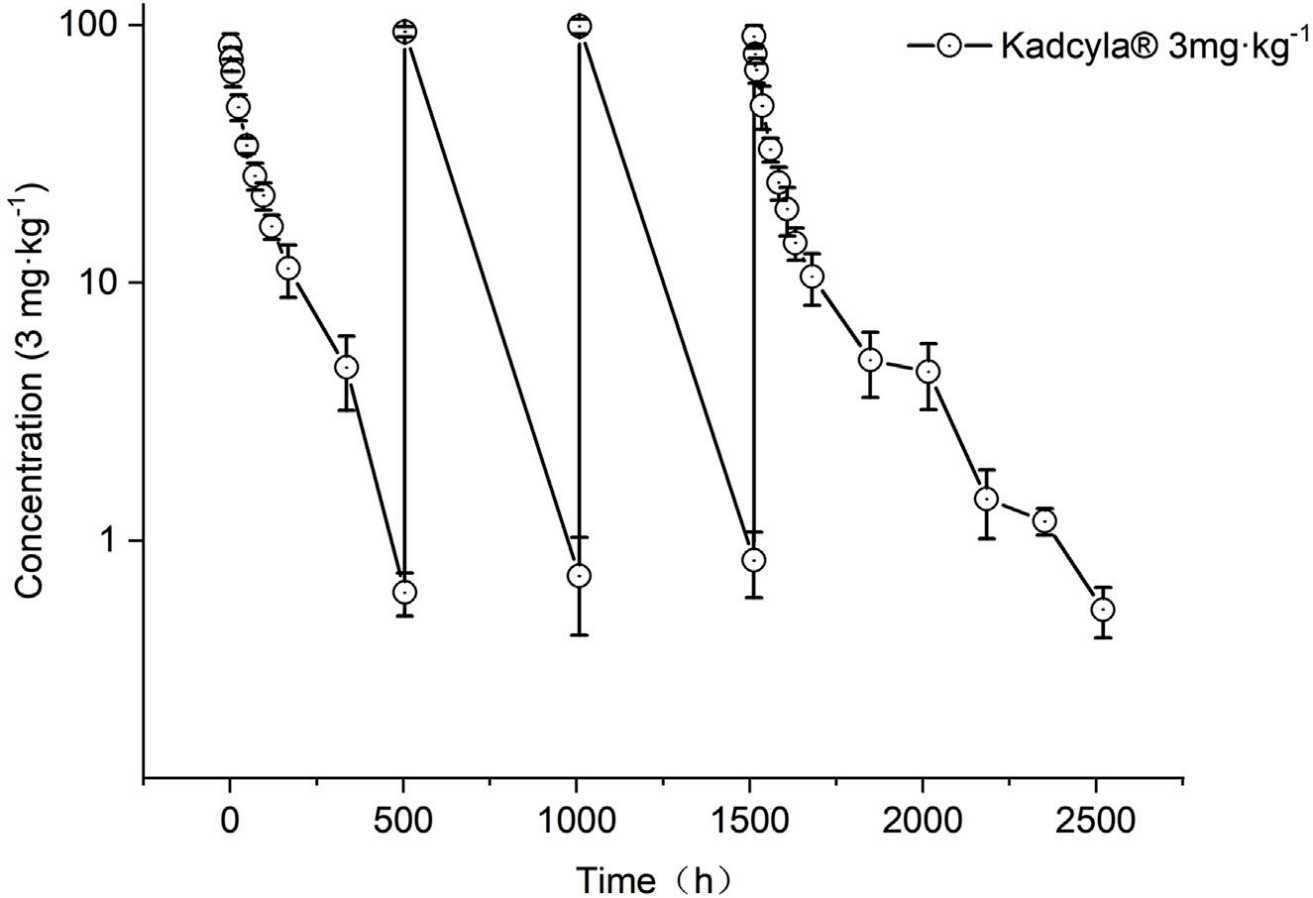
ADC serum concentration time curves of HS630 3 mg·kg^−1^ after continuous intravenous infusion in cynomolgus monkeys.

The ADC concentration–time curve at administration is shown in Figure 9. Table 7 indicates the ADC pharmacokinetic parameters calculated after the first and last administration of HS630 3 mg·kg^−1^. There was no statistically significant difference in the comparison of AUC_(0–504 h)_ and C_max_ between the first and last doses, with an accumulation factor of 1.04 ± 0.20. The serum concentration of ADC in the first and last doses is summarized for comparison in Table 8.

**TABLE 7:** Comparison of ADC pharmacokinetic parameters between the first and last doses after continuous intravenous infusion of HS630 3 mg·kg^−1^ in cynomolgus monkeys.

Parameter	Unit	First dose	Last dose	T-test *p*-value
AUC_(0–504 h)_	μg·h·mL^−1^	6,692.88 ± 806.01	6,837.80 ± 1,047.5	0.79
C_max_	μg·mL^−1^	83.43 ± 9.03	90.44 ± 9.08	0.21
T_max_	h	0.5 ± 0	0.5 ± 0	--
Accumulation factors			1.04 ± 0.20	--

**TABLE 8:** Comparison of ADC serum concentration between the first and last doses after continuous intravenous infusion of HS630 3 mg·kg^−1^ in cynomolgus monkeys (n = 6).

Time (h)	Concentration (µg·mL^−1^)	
	Continuous dosing group (first dose)	Continuous dosing group (last dose)
0 (1,512)	ND***	0.84 ± 0.24
0.5 (1,512.5)	83.07 ± 9.40	90.44 ± 9.08
4 (1,516)	73.8 ± 7.91	77.18 ± 6.11
8 (1,520)	65.63 ± 8.05	66.98 ± 7.48
24 (1,536)	48.04 ± 5.54	48.65 ± 9.22
48 (1,560)	34.06 ± 2.39	32.95 ± 3.55
72 (1,584)	25.98 ± 3.07	24.50 ± 3.61
96 (1,608)	21.82 ± 2.65	19.33 ± 4.09
120 (1,632)	16.55 ± 1.78	14.29 ± 2.05
168 (1,680)	11.39 ± 2.62	10.59 ± 2.41
336 (1848)	4.70 ± 1.50	5.02 ± 1.42
504 (2016)	0.63 ± 0.12**	4.52 ± 1.29
2,184		1.45 ± 0.43
2,352		1.19 ± 0.14
2,520		0.54 ± 0.12

*p < 0.05, **p < 0.01, and ***p < 0.001: significant difference by paired t-test compared to the last dose group.

#### 3.4.3 Comparison of ADC pharmacokinetic parameters after single intravenous infusion of 3 mg·kg^−1^ HS630 and Kadcyla® in cynomolgus monkeys

**FIGURE 10:**
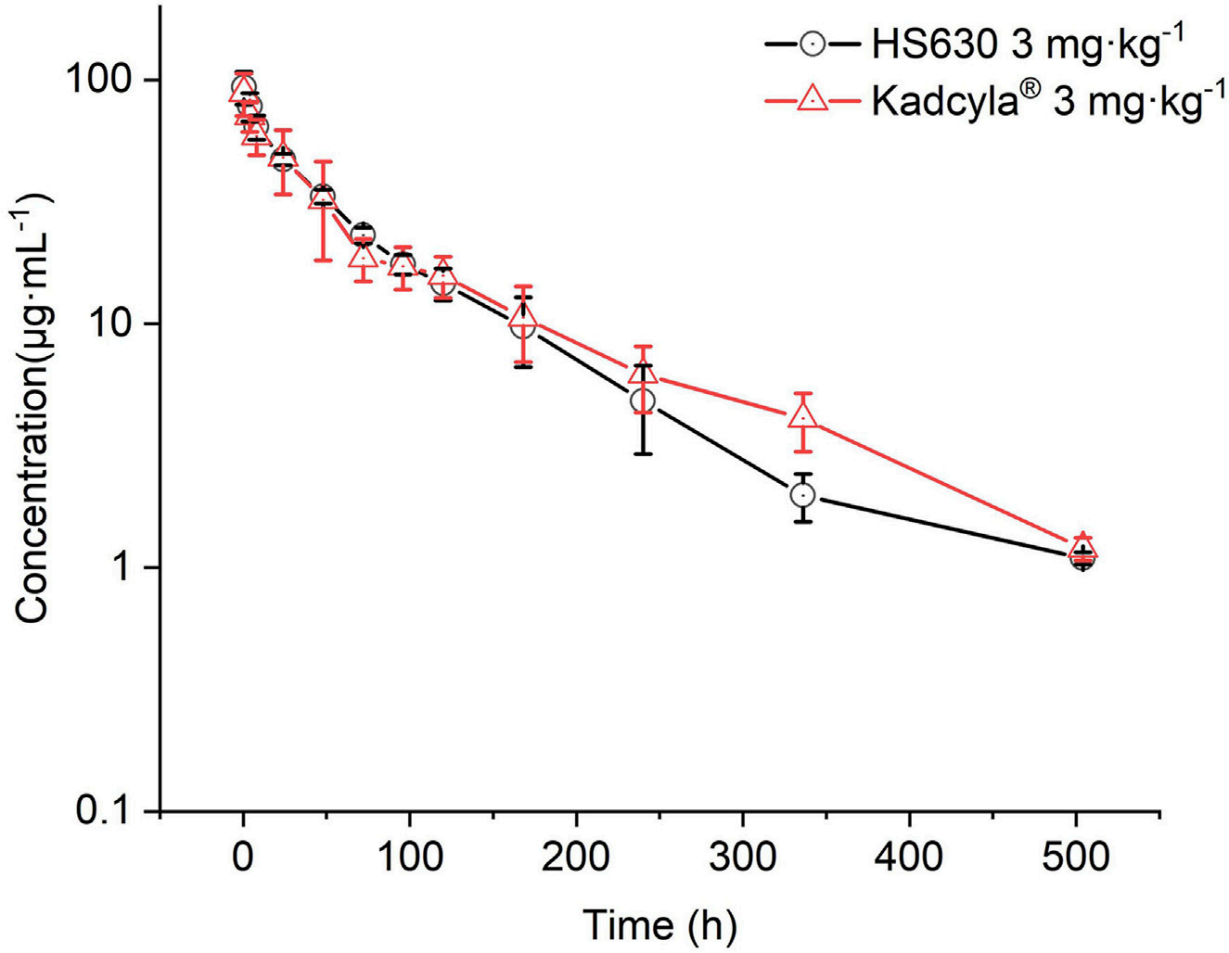
Comparison of ADC serum concentration–time curves after single intravenous infusion of 3 mg·kg^−1^ HS630 and Kadcyla® in cynomolgus monkeys.

Figure 10 presents the results for comparing the ADC concentration–time curve in pharmacokinetics between HS630 and control drugs of the same dose, showing almost complete coincidence in the two curves. Table 9 reveals the pharmacokinetic parameters after a 3 mg·kg^−1^ single intravenous infusion of the control drug Kadcyla® in cynomolgus monkeys. The post-administration T_max_ of ADC was also 0.5 h, followed by a slower elimination with a detectable serum drug concentration of 504 h. T_1/2_ after a single administration of the 3 mg·kg^−1^ HS630 and Kadcyla® were 101.68 ± 8.05 h and 107.78 ± 5.99 h; AUC_(0–504 h)_ was 5,712.66 ± 528.5 μg·h·mL^−1^ and 6,007.31 ± 954.41 μg·h·mL^−1^; CL was 0.51 ± 0.05 mL·kg^−1^·h^−1^ and 0.49 ± 0.07 mL·kg^−1^·h^−1^; and C_max_ was 93.56 ± 14.21 μg·mL^−1^ and 88.50 ± 17.43 μg·mL^−1^, respectively. WinNonlin was used for biosimilarity assessment HS630 and Kadcyla®. The 90% confidence intervals (CIs) of the geometric mean ratio of C_max_ and AUC_(0–t)_ were 109.15% and 97.51%, respectively.

**TABLE 9:** Comparison of ADC pharmacokinetic parameters after single intravenous infusion of 3 mg·kg^−1^ HS630 and Kadcyla^®^ in cynomolgus monkeys.

Parameter	Unit	HS630 3 mg·kg^−1^	Kadcyla® 3 mg·kg^−1^	T-test *p-*value
AUC_(0–504 h)_	μg·h·mL^−1^	5,712.66 ± 528.5	6,007.31 ± 954.41	0.527
AUC_(0–inf)_	μg·h·mL^−1^	5,872.89 ± 521.13	6,194.25 ± 946.77	0.488
AUC_(504 h–inf)_	μg·h·mL^−1^	160.23 ± 9.74	186.94 ± 27	0.061
MRT	h	94.08 ± 8.45	112.29 ± 11.32	0.011*
CL	ml·kg^−1^·h^−1^	0.51 ± 0.05	0.49 ± 0.07	0.575
V_ss_	mL·kg^−1^	48.08 ± 1.35	55.63 ± 11.17	0.159
T_1/2_	h	101.68 ± 8.05	107.78 ± 5.99	0.170
Kel	h^−1^	0.01 ± 0	0.01 ± 0	0.156
C_max_	μg·mL^−1^	93.56 ± 14.21	88.50 ± 17.43	0.064
T_max_	h	0.5 ± 0	0.5 ± 0	--

^*^ represents *p* < 0.05; ** represents *p* < 0.01; *** represents *p* < 0.001.

#### 3.4.4 Pharmacokinetic parameters of total antibody after single intravenous infusion of different doses of HS630 in cynomolgus monkeys

**FIGURE 11:**
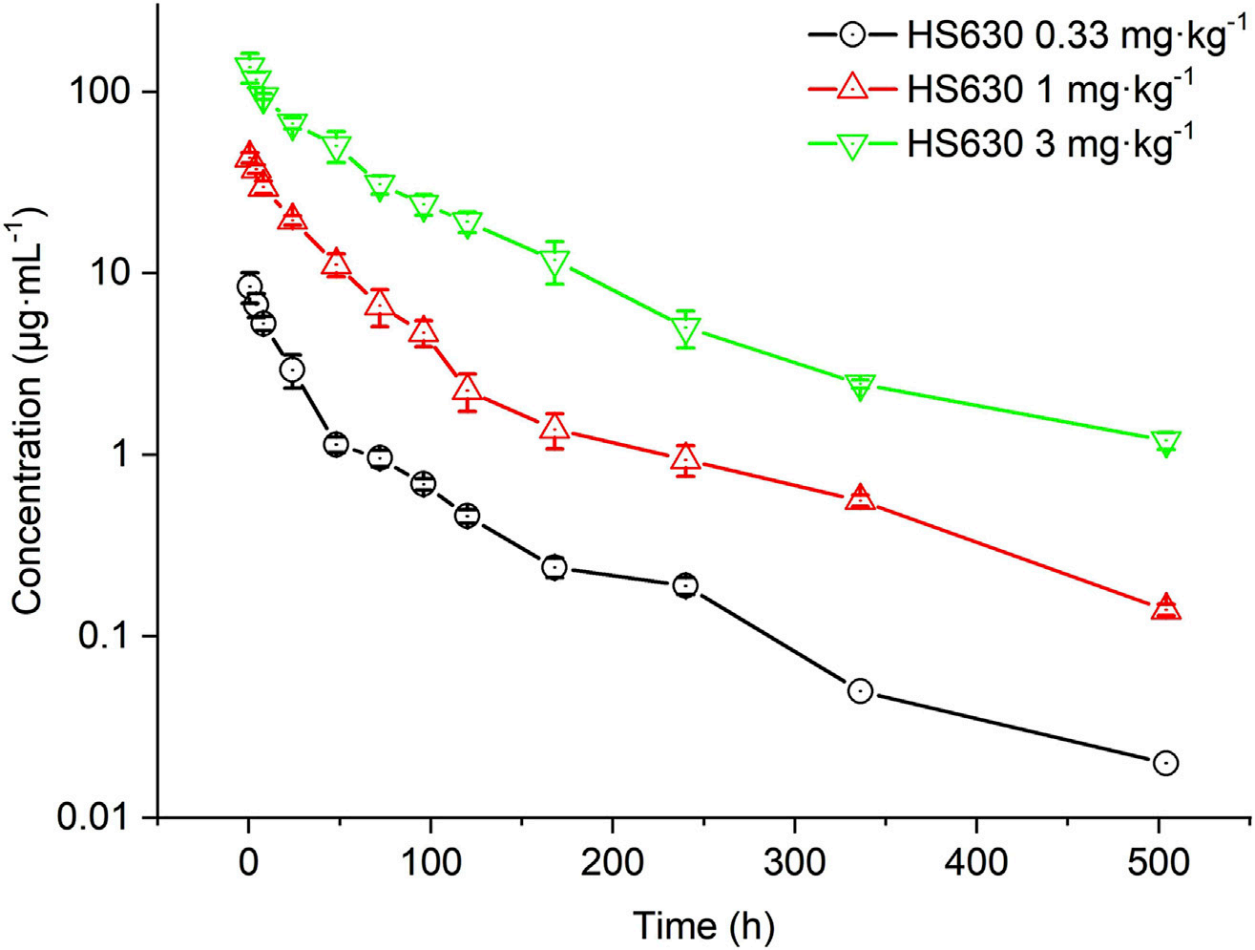
Total antibody serum concentration–time curves of HS630 after single intravenous infusion at different doses in cynomolgus monkeys.

The concentrations of total antibody in the serum of cynomolgus monkeys after a single intravenous infusion of HS630 0.33 mg·kg^−1^, 1 mg·kg^−1^, and 3 mg·kg^−1^ is shown in Figure 11. Table 10 summarizes the total antibody pharmacokinetic parameters of HS630 at these doses. The peak times were all at 0.5 h after administration, followed by slower elimination, and the serum drug concentrations were detectable up to 504 h for all three doses. Terminal phase half-lives T_1/2_ were 87.12 ± 3.79 h, 100.55 ± 10.11 h, and 97.08 ± 8.29 h, respectively. AUC_(0–504 h)_ was 276.24 ± 19.08 μg·h·mL^−1^, 1781.05 ± 156.35 μg·h·mL^−1^, and 7,770.31 ± 514.83 μg·h·mL^−1^, respectively, revealing statistically significant differences among the three groups (*p* < 0.01). CL was 1.19 ± 0.08 mL·kg^−1^·h^−1^, 0.56 ± 0.05 mL·kg^−1^·h^−1^, and 0.38 ± 0.02 mL·kg^−1^·h^−1^, respectively. T_max_ was all at 0.5 h after administration, and C_max_ was 8.45 ± 1.63 μg·mL^−1^, 43.25 ± 2.88 μg·mL^−1^, and 142.80 ± 19.21 μg·mL^−1^, respectively.

**TABLE 10:** Pharmacokinetic parameters of total antibody after single intravenous infusion of different doses of HS630 in cynomolgus monkeys.

Parameter	Unit	Low dose (0.33 mg·kg^−1^)	Medium dose (1 mg·kg^−1^)	High dose (3 mg·kg^−1^)
AUC_(0–5044 h)_	μg·h·mL^−1^	276.24 ± 19.08	1781.05 ± 156.35	7,770.31 ± 514.83
AUC_(0–inf)_	μg·h·mL^−1^	279.05 ± 19.09	1800.7 ± 155.9	7,938.88 ± 496.02
AUC_(504 h–inf)_	μg·h·mL^−1^	2.81 ± 0.57	19.65 ± 3.22	168.58 ± 25.60
MRT	h	65.13 ± 3.73	67.17 ± 2.98	86.34 ± 5.09
CL	mL·kg^−1^·h^−1^	1.19 ± 0.08	0.56 ± 0.05	0.38 ± 0.02
V_ss_	mL·kg^−1^	77.55 ± 9.39	37.49 ± 2.9	32.67 ± 1.78
T_1/2_	h	87.12 ± 3.79	100.55 ± 10.11	97.08 ± 8.29
Kel	h^−1^	0.01 ± 0	0.01 ± 0	0.01 ± 0
C_max_	μg·mL^−1^	8.45 ± 1.63	43.26 ± 2.88	142.80 ± 19.21
T_max_	h	0.5 ± 0	0.5 ± 0	0.5 ± 0

#### 3.4.5 Pharmacokinetic parameters of total antibody after continuous intravenous administrations of high-dose HS630 in cynomolgus monkeys

**FIGURE 12:**
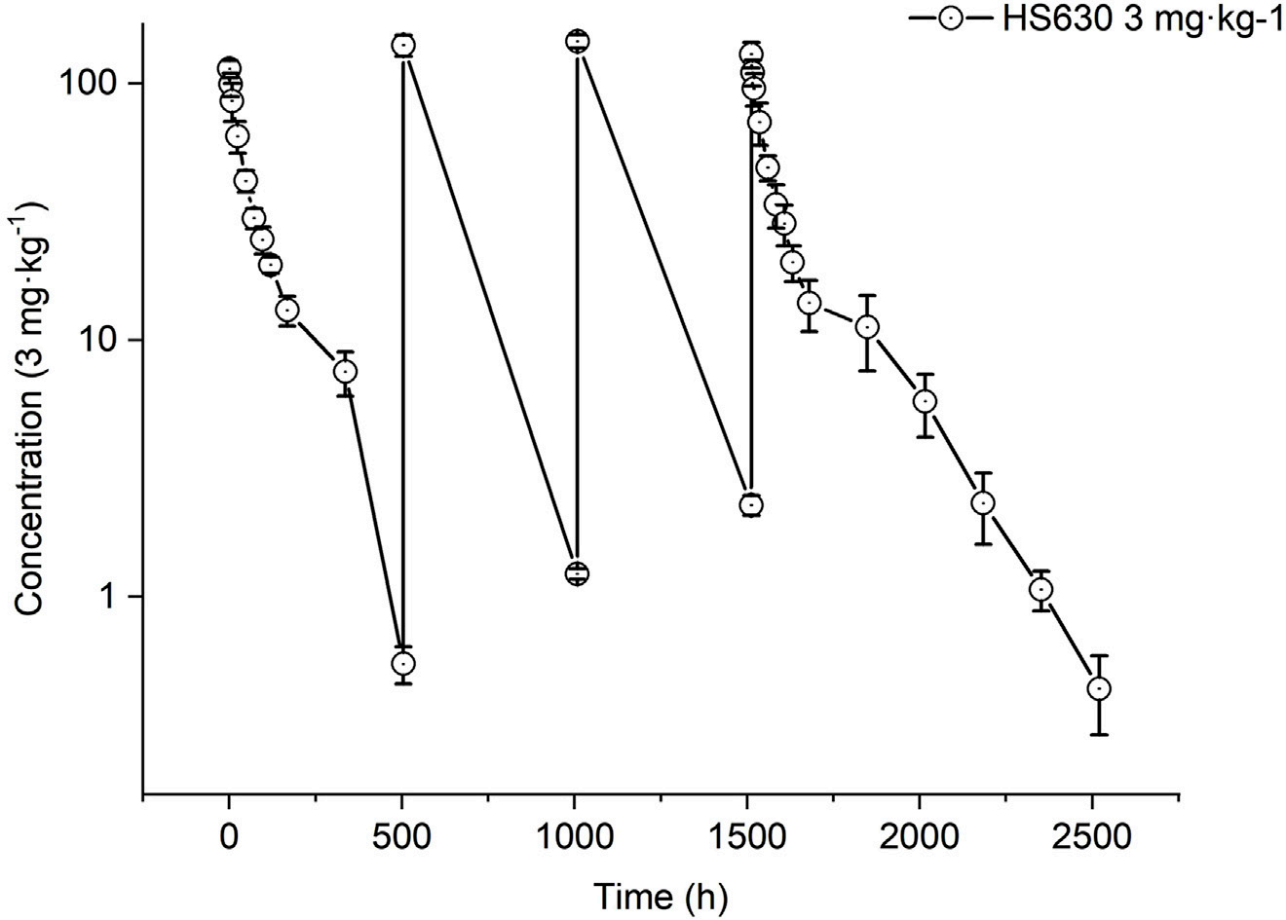
Total antibody serum concentration–time curves of HS630 3 mg·kg^−1^ after continuous intravenous infusion in cynomolgus monkeys.

The total antibody concentration–time curve is plotted in Figure 12 in cynomolgus monkeys after continuous intravenous infusion of 3 mg·kg^−1^ HS630. Table 11 shows the total antibody pharmacokinetic parameters calculated after the first and last administration. The AUC _(0–504 h)_ and C_max_ of total antibody exhibited no statistically significant difference in the comparison between the first and last doses, and the accumulation factor was 1.22 ± 0.18. The concentrations of total antibody in serum at the first and last doses are summarized in Table 12.

**TABLE 11:** Comparison of total antibody pharmacokinetic parameters between the first and last doses after continuous intravenous infusion of 3 mg·kg^−1^ HS630 in cynomolgus monkeys.

Parameter	Unit	First dose	Last dose	T-test *p*-value
AUC_(0–504 h)_	μg·h·mL^−1^	8,440.15 ± 813.20	10268.38 ± 1811.87	0.059
C_max_	μg·mL^−1^	113.98 ± 8.87	129.78 ± 14.78	0.054
T_max_	h	0.5 ± 0	0.5 ± 0	--
Accumulation factors			1.22 ± 0.18	--

**TABLE 12:** Comparison of the total antibody serum concentration between the first and last doses after continuous intravenous infusion of HS630 3 mg·kg^−1^ in cynomolgus monkeys (n = 6).

Time (h)	Concentration (µg·mL−1)	
	Continuous dosing group (first dose)	Continuous dosing group (last dose)
0 (1,512)	ND***	2.28 ± 0.2
0.5 (1,512.5)	113.98 ± 8.87	129.78 ± 14.78
4 (1,516)	99.34 ± 10.34	110.41 ± 12.82
8 (1,520)	85.42 ± 14.34	95.4 ± 13.68
24 (1,536)	62.21 ± 8.83	70.61 ± 13.23
48 (1,560)	41.74 ± 4.03	47.02 ± 5.28
72 (1,584)	29.85 ± 2.70	33.77 ± 6.49
96 (1,608)	24.62 ± 2.94	28.42 ± 5.09
120 (1,632)	19.62 ± 1.47	20.08 ± 3.21
168 (1,680)	13.08 ± 1.71	13.96 ± 3.15
336 (1848)	7.54 ± 1.47	11.25 ± 3.66
504 (2016)	0.55 ± 0.09***	5.78 ± 1.59
2,184		2.32 ± 0.72
2,352		1.7 ± 1.69
2,520		0.65 ± 0.41

*p < 0.05, **p < 0.01, ***, *p* < 0.001: significant difference by paired t-test compared to the last dose group.

#### 3.4.6 Comparison of total antibody pharmacokinetic parameters after single intravenous infusion of 3 mg·kg^−1^ HS630 and Kadcyla® in cynomolgus monkeys

**FIGURE 13:**
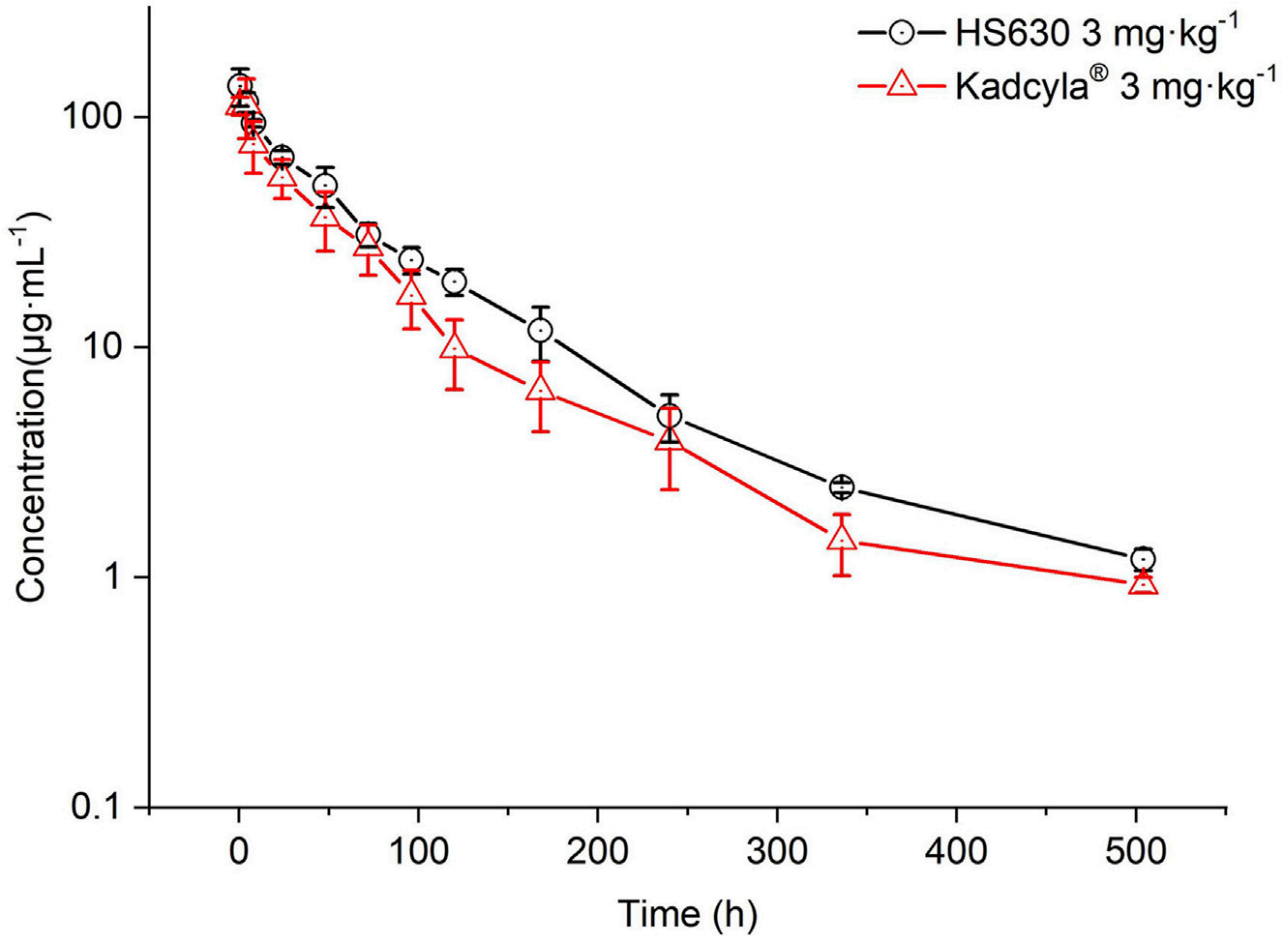
Comparison of total antibody serum concentration–time curves after single intravenous infusion of 3 mg·kg^−1^ HS630 and Kadcyla® in cynomolgus monkeys.

The comparison of total antibody concentration–time curve in serum concentration between HS630 and control drugs at the same dose is shown in Figure 13 and Table 13. Table 14 is the total antibody pharmacokinetic parameters after a 3 mg·kg^−1^ single intravenous infusion of the control drug Kadcyla® in cynomolgus monkeys. T_max_ of total antibodies was also 0.5 h after administration, followed by slow elimination, and the serum drug concentration could be detected to 504 h. T_1/2_ of 3 mg·kg^−1^ control drug was 112.72 ± 12.46 h, AUC_(0–504 h)_ was 5,789.06 ± 705.97 μg·h·mL^−1^ after a single administration, CL was 0.51 ± 0.05 mL·kg^−1^·h^−1^, and C_max_ was 126.14 ± 24.18 μg·mL^−1^. WinNonlin was used for biosimilarity assessment HS630 and Kadcyla®. The 90% CI of the geometric mean ratio of the total antibody C_max_ and _AUC(0–t)_ was 116.23% and 136.16%, respectively.

**TABLE 13:** Comparison of the total antibody serum concentration after single intravenous infusion of 3 mg·kg^−1^ HS630 and Kadcyla^®^ in cynomolgus monkeys.

Time(h)	Serum drug concentrations (µg·mL^−1^)	
	HS630(3 mg·kg^−1^)	Kadcyla®(3 mg·kg^−1^)
0	ND	ND
0.5	136.77 ± 25.33	112.15 ± 10.04
4	116.34 ± 11.50	113.58 ± 32.93
8	94.02 ± 3.44	76.42 ± 19.42
24	67.03 ± 4.55	54.74 ± 10.43
48	50.49 ± 9.88	36.70 ± 10.56
72	30.89 ± 3.57	27.25 ± 6.71
96	23.97 ± 3.13	16.80 ± 4.78
120	19.26 ± 2.50	9.86 ± 3.29
168	11.83 ± 3.13	6.47 ± 2.17
240	5.04 ± 1.16	3.92 ± 1.52
336	2.46 ± 0.13	1.45 ± 0.43
504	1.2 ± 0.13	0.93 ± 0.07

**TABLE 14:** Comparison of total antibody pharmacokinetic parameters between the first and last doses after continuous intravenous infusion of 3 mg·kg^−1^ HS630 and Kadcyla^®^ in cynomolgus monkeys.

Parameter	Unit	HS630 3 mg·kg^−1^	Kadcyla® 3 mg·kg^−1^	T-test *p*-value
AUC_(0–504 h)_	μg·h·mL^−1^	7,770.31 ± 514.83	5,789.06 ± 705.97	0.000***
AUC_(0–inf)_	μg·h·mL^−1^	7,938.88 ± 496.02	5,940.8 ± 696.92	0.000***
AUC_(504 h–inf)_	μg·h·mL^−1^	168.58 ± 25.60	151.74 ± 27.30	0.296
MRT	h	86.34 ± 5.09	78.03 ± 4.34	0.013*
CL	ml·kg^−1^·h^−1^	0.38 ± 0.02	0.51 ± 0.05	0.001**
V_ss_	mL·kg^−1^	32.67 ± 1.78	39.73 ± 3.86	0.005**
T_1/2_	h	97.08 ± 8.29	112.72 ± 12.46	0.031*
Kel	h^−1^	0.01 ± 0	0.01 ± 0	0.031*
C_max_	μg·mL^−1^	142.80 ± 19.21	126.14 ± 24.18	0.217
T_max_	h	0.5 ± 0	0.5 ± 0	--

^*^ represents *p* < 0.05; ** represents *p* < 0.01; *** represents *p* < 0.0014.

#### 3.4.7 Results of DM1 concentration determination in the serum samples of cynomolgus monkeys

Data for DM1 concentration in serum are summarized in Table 15 without further statistical analysis. It highlights that the HS630 conjugated drug was stable and not easily broken in the blood.

**TABLE 15:** Comparison of DM1 serum concentration after single intravenous infusion of HS630 and Kadcyla^®^ in cynomolgus monkeys.

Time (h)	HS630						Kadcyla®	
	0.33 mg·kg^−1^		1 mg·kg^−1^		3 mg·kg^−1^		3 mg·kg^−1^	
	Concentration (ng·mL^−1^)							
	Mean	Standard deviation	Mean	Standard deviation	Mean	Standard deviation	Mean	Standard deviation
0.5	0.55	0.50	2.31	0.62	5.39	0.72	3.00	0.88
4	ND	ND	0.77	0.13	1.43	0.24	1.01	0.15
8	ND	ND	ND	ND	0.77	0.13	0.59	0.07
24	ND	ND	ND	ND	ND	ND	ND	ND
48	ND	ND	ND	ND	ND	ND	ND	ND
72	ND	ND	ND	ND	ND	ND	ND	ND
96	ND	ND	ND	ND	ND	ND	ND	ND
120	ND	ND	ND	ND	ND	ND	ND	ND
168	ND	ND	ND	ND	ND	ND	ND	ND
240	ND	ND	ND	ND	ND	ND	ND	ND
336	ND	ND	ND	ND	ND	ND	ND	ND
504	ND	ND	ND	ND	ND	ND	ND	ND

### 3.5 Immunogenicity in cynomolgus monkeys

#### 3.5.1 Positive judgment value

**FIGURE 14:**
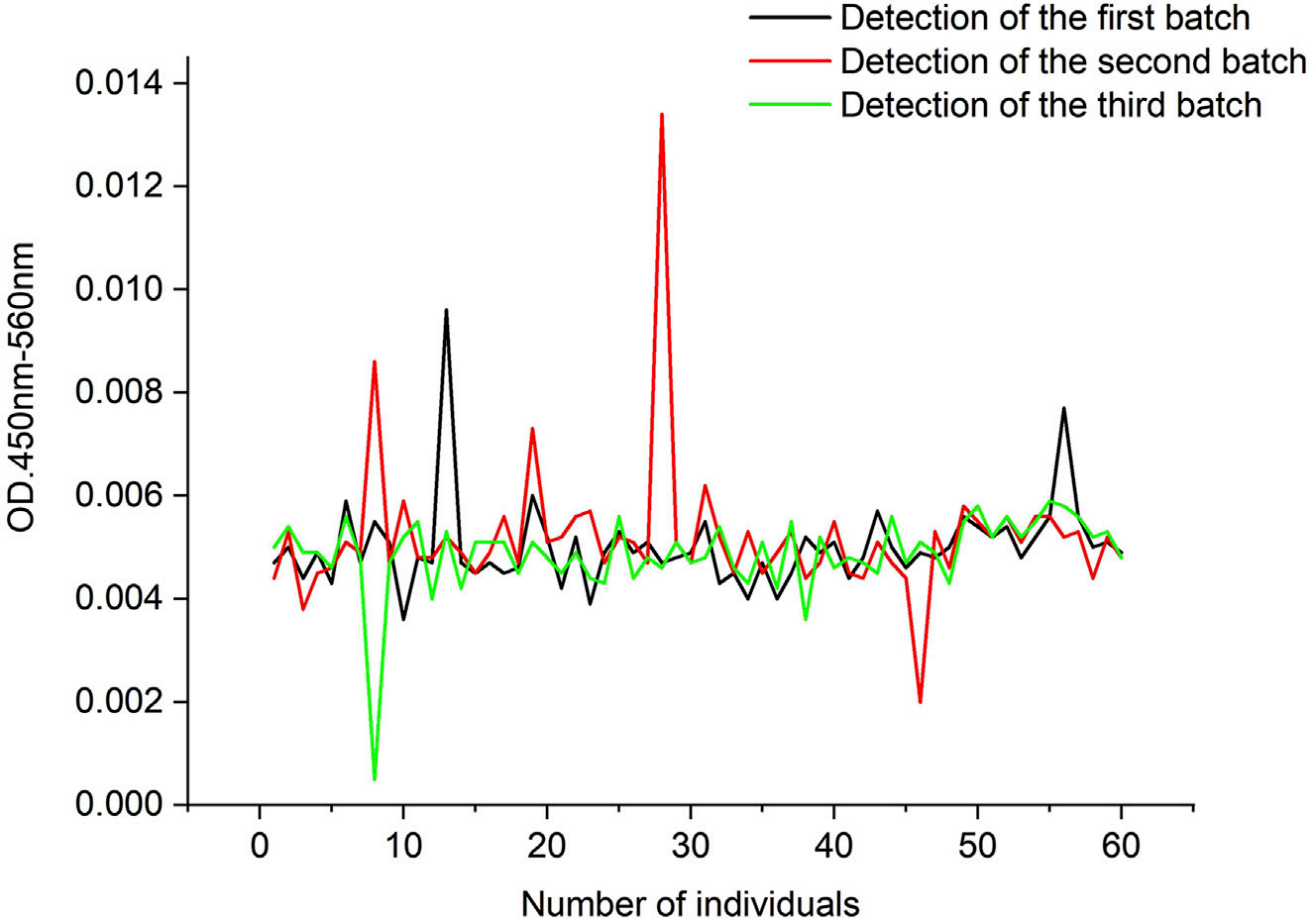
Fluctuations of OD values for different assay batches of 60 cynomolgus monkey serum samples.

As indicated in Table 16 and Figure 14, the cut-off values of the three batches were 0.0076, 0.0092, and 0.0071, respectively, and their mean value was 0.0080. As a result, the positive judgment value was set as 0.0080, with the positive result determined for samples with a value higher than 0.0080.

**TABLE 16:** Data for critical value determination.

Monkey No	Detection of the first batch	Detection of the second batch	Detection of the third batch
1	0.0047	0.0044	0.0050
2	0.0050	0.0053	0.0054
3	0.0044	0.0038	0.0049
4	0.0049	0.0045	0.0049
5	0.0043	0.0046	0.0046
6	0.0059	0.0051	0.0056
7	0.0047	0.0049	0.0048
8	0.0055	0.0086	0.0005
9	0.0051	0.0047	0.0047
10	0.0036	0.0059	0.0052
11	0.0048	0.0048	0.0055
12	0.0047	0.0048	0.0040
13	0.0096	0.0052	0.0053
14	0.0047	0.0049	0.0042
15	0.0045	0.0045	0.0051
16	0.0047	0.0049	0.0051
17	0.0045	0.0056	0.0051
18	0.0046	0.0047	0.0045
19	0.0060	0.0073	0.0051
20	0.0052	0.0051	0.0048
21	0.0042	0.0052	0.0045
22	0.0052	0.0056	0.0049
23	0.0039	0.0057	0.0044
24	0.0049	0.0047	0.0043
25	0.0053	0.0052	0.0056
26	0.0049	0.0051	0.0044
27	0.0051	0.0047	0.0048
28	0.0047	0.0134	0.0046
29	0.0048	0.0051	0.0051
30	0.0049	0.0047	0.0047
31	0.0055	0.0062	0.0048
32	0.0043	0.0052	0.0054
33	0.0045	0.0045	0.0046
34	0.0040	0.0053	0.0043
35	0.0047	0.0045	0.0051
36	0.0040	0.0049	0.0042
37	0.0045	0.0053	0.0055
38	0.0052	0.0044	0.0036
39	0.0049	0.0047	0.0052
40	0.0051	0.0055	0.0046
41	0.0044	0.0045	0.0048
42	0.0048	0.0044	0.0047
43	0.0057	0.0051	0.0045
44	0.0050	0.0047	0.0056
45	0.0046	0.0044	0.0047
46	0.0049	0.0020	0.0051
47	0.0048	0.0053	0.0049
48	0.0050	0.0046	0.0043
49	0.0056	0.0058	0.0055
50	0.0054	0.0055	0.0058
51	0.0052	0.0052	0.0052
52	0.0054	0.0056	0.0056
53	0.0048	0.0051	0.0052
54	0.0052	0.0056	0.0055
55	0.0056	0.0056	0.0059
56	0.0077	0.0052	0.0058
57	0.0056	0.0053	0.0056
58	0.0050	0.0044	0.0052
59	0.0051	0.0052	0.0053
60	0.0049	0.0048	0.0048
Mean	0.0050	0.0052	0.0049
Standard deviation	0.0009	0.0014	0.0008
Critical value	0.0076	0.0092	0.0071

#### 3.5.2 Results of bridge ELISA assay

In Tables 17–21, through double-antibody sandwich ELISA, the test values of samples 4 # (0.5 h), 8 # (96 h), 13 # (24 h), 17 # (504 h), 20 # (1,008.5 h and 1,512.5 h), 22 # (1,512.5 h), 24 # (1,008.5 h and 1,512.5 h), and 28 # (48 h) were higher than the cut-off value. The samples required further confirmation, and other samples did not produce anti-drug antibody.

**TABLE 17:** Anti-HS630 antibody detection of pharmacokinetic serum samples after single intravenous infusion of 0.33 mg·kg^−1^ HS630.

Blood collection time (h)	1#	2#	3#	4#	5#	6#
0	0.0030	0.0030	0.0039	0.0026	0.0027	0.0030
0.5	0.0037	0.0037	0.0038	0.0199	0.0029	0.0045
4	0.0033	0.0041	0.0040	0.0025	0.0031	0.0035
8	0.0032	0.0042	0.0038	0.0047	0.0029	0.0037
24	0.0043	0.0038	0.0033	0.0034	0.0029	0.0037
48	0.0033	0.0035	0.0031	0.0028	0.0029	0.0039
72	0.0049	0.0033	0.0032	0.0025	0.0024	0.0032
96	0.0038	0.0034	0.0033	0.0040	0.0028	0.0034
120	0.0037	0.0037	0.0033	0.0026	0.0034	0.0026
168	0.0034	0.0034	0.0032	0.0033	0.0036	0.0034
240	0.0039	0.0036	0.0030	0.0033	0.0035	0.0039
336	0.0032	0.0036	0.0027	0.0031	0.0034	0.0041
504	0.0033	0.0035	0.0076	0.0029	0.0043	0.0037

**TABLE 18:** Anti-HS630 antibody detection of pharmacokinetic serum samples after single intravenous infusion of 1 mg·kg^−1^ HS630.

Blood collection time (h)	7#	8#	9#	10#	11#	12#
0	0.0035	0.0029	0.0026	0.0030	0.0038	0.0035
0.5	0.0032	0.0034	0.0024	0.0026	0.0032	0.0033
4	0.0032	0.0033	0.0023	0.0027	0.0033	0.0035
8	0.0037	0.0035	0.0025	0.0031	0.0032	0.0036
24	0.0035	0.0030	0.0029	0.0034	0.0039	0.0039
48	0.0034	0.0026	0.0030	0.0037	0.0041	0.0043
72	0.0035	0.0025	0.0033	0.0013	0.0042	0.0035
96	0.0036	0.0080	0.0030	0.0045	0.0039	0.0032
120	0.0037	0.0026	0.0030	0.0033	0.0031	0.0031
168	0.0040	0.0023	0.0028	0.0047	0.0034	0.0035
240	0.0044	0.0024	0.0034	0.0037	0.0035	0.0031
336	0.0037	0.0041	0.0027	0.0038	0.0036	0.0037
504	0.0029	0.0027	0.0030	0.0036	0.0034	0.0028

**TABLE 19:** Anti-HS630 antibody detection of pharmacokinetic serum samples after single intravenous infusion of 3 mg·kg^−1^ HS630.

Blood collection time (h)	13#	14#	15#	16#	17#	18#
0	0.0022	0.0030	0.0035	0.0041	0.0045	0.0023
0.5	0.0026	0.0031	0.0032	0.0042	0.0036	0.0047
4	0.0079	0.0030	0.0044	0.0039	0.0030	−0.0003
8	0.0026	0.0030	0.0032	0.0032	0.0026	0.0026
24	0.0140	0.0029	0.0048	0.0032	0.0033	0.0023
48	0.0024	0.0031	0.0035	0.0034	0.0033	0.0021
72	0.0052	0.0030	0.0039	0.0035	0.0033	0.0025
96	0.0004	0.0031	0.0036	0.0035	0.0029	0.0064
120	0.0026	0.0031	0.0039	0.0034	0.0046	0.0033
168	0.0025	0.0028	0.0031	0.0033	0.0025	0.0033
240	0.0021	0.0027	0.0035	0.0033	0.0077	0.0030
336	0.0025	0.0032	0.0031	0.0040	0.0026	0.0032
504	0.0028	0.0034	0.0037	0.0039	0.0688	0.0027

**TABLE 20:** Anti-HS630 antibody detection of pharmacokinetic serum samples after continuous intravenous infusion of 3 mg·kg^−1^ HS630.

Blood collection time (h)	19#	20#	21#	22#	23#	24#
0	0.0032	0.0033	0.0032	0.0031	0.0029	0.0032
0.5	0.0024	0.0036	0.0017	0.0032	0.0028	0.0035
4	0.0029	0.0039	0.0027	0.0038	0.0029	0.0040
8	0.0029	0.0041	0.0027	0.0040	0.0029	0.0038
24	0.0027	0.0042	0.0028	0.0043	0.0027	0.0044
48	0.0028	0.0036	0.0028	0.0035	0.0027	0.0035
72	0.0032	0.0032	0.0032	0.0032	0.0030	0.0033
96	0.0038	0.0031	0.0034	0.0029	0.0034	0.0031
120	0.0036	0.0033	0.0034	0.0031	0.0034	0.0034
168	0.0036	0.0033	0.0032	0.0032	0.0034	0.0030
336	0.0045	0.0034	0.0044	0.0033	0.0043	0.0035
504	0.0034	0.0030	0.0029	0.0030	0.0028	0.0031
504.5	0.0048	0.0029	0.0047	0.0026	0.0048	0.0030
1,008	0.0038	0.0028	0.0036	0.0027	0.0038	0.0027
1,008.5	0.0037	0.0084	0.0034	0.0077	0.0036	0.0088
1,512	0.0036	0.0026	0.0035	0.0024	0.0033	0.0025
1,512.5	0.0040	0.0776	0.0039	0.0185	0.0040	0.0777
1,516	0.0033	0.0025	0.0031	0.0024	0.0030	0.0025
1,520	0.0035	0.0051	0.0035	0.0050	0.0037	0.0045
1,536	0.0031	0.0063	0.0028	0.0031	0.0029	0.0025
1,560	0.0039	0.0027	0.0037	0.0028	0.0036	0.0029
1,584	0.0041	0.0025	0.0041	0.0024	0.0041	0.0024
1,608	0.0042	0.0015	0.0040	0.0021	0.0041	0.0018
1,632	0.0039	0.0025	0.0036	0.0026	0.0039	0.0026
1,680	0.0036	0.0058	0.0035	0.0065	0.0036	0.0069
1848	0.0036	0.0032	0.0031	0.0032	0.0033	0.0032
2016	0.0037	0.0032	0.0034	0.0031	0.0034	0.0032
2,184	0.0037	0.0028	0.0035	0.0029	0.0035	0.0029
2,352	0.0036	0.0028	0.0034	0.0028	0.0034	0.0028
2,520	0.0035	0.0028	0.0034	0.0028	0.0039	0.0026

**TABLE 21:** Anti-HS630 antibody detection of pharmacokinetic serum samples after single intravenous infusion of 3 mg·kg^−1^ Kadcyla^®^.

Blood collection time (h)	25#	26#	27#	28#	29#	30#
0	0.0033	0.0036	0.0033	0.0030	0.0021	0.0027
0.5	0.0045	0.0036	0.0033	0.0033	0.0025	0.0033
4	0.0029	0.0034	0.0035	0.0030	0.0032	0.0037
8	0.0030	0.0041	0.0035	0.0028	0.0030	0.0033
24	0.0027	0.0030	0.0034	0.0027	0.0035	0.0033
48	0.0025	0.0033	0.0034	0.0081	0.0028	0.0043
72	0.0031	0.0028	0.0037	0.0025	0.0029	0.0031
96	0.0034	0.0037	0.0039	0.0023	0.0028	0.0049
120	0.0034	0.0040	0.0029	0.0024	0.0032	0.0038
168	0.0034	0.0042	0.0037	0.0050	0.0029	0.0037
240	0.0042	0.0038	0.0034	−0.0024	0.0030	0.0036
336	0.0032	0.0035	0.0029	0.0029	0.0027	0.0040
504	0.0049	0.0034	0.0031	0.0024	0.0027	0.0032

#### 3.5.3 Corroboration: immune clearance

The immune confirmation results are shown at Table 22. There was no significant decrease in the signal value of the samples after immune clearance of 4# (0.5 h), 8# (96 h), 13# (24 h), 17# (504 h), 20# (1,008.5 h and 1,512.5 h), 22# (1,512.5 h), 24# (1,008.5 h and 1,512.5 h), and 28# (48H). The samples were false-positive, with the high signal value being attributed to the impact of the serum matrix.

**TABLE 22:** Results of immune confirmation.

Sample No	10% serum dilution	High concentration drug product dilution
4# (0.5 h)	0.0119	0.0127
8# (96 h)	0.0112	0.0112
13# (24 h)	0.0120	0.0152
17# (504 h)	0.0132	0.0154
20# (1,008.5 h)	0.0142	0.0230
20# (1,512.5 h)	0.0177	0.0251
22# (1,512.5 h)	0.0119	0.0157
24# (1,008.5 h)	0.0122	0.0143
24# (1,512.5 h)	0.0119	0.0151
28# (48 h)	0.0126	0.0173

## 4 Discussion

HS630 is a proposed T-DM1 biosimilar of Kadcyla®. Through *in vitro* and *in vivo* experiments, this study intended to demonstrate the similarity between HS630 and Kadcyla®. Biosimilars of such ADCs are critical in improving global access to targeted cancer therapies, but their approval hinges on a rigorous demonstration of similarity to the originator. The intracellular trafficking of T-DM1 usually relies on binding to the HER2 on the plasma membrane, and the HER2–T-DM1 complex enters cells via receptor-mediated endocytosis (Ritchie et al., 2013; Barok et al., 2014). Therefore, the tumor-specific delivery of cytotoxic payloads depends largely on high-affinity binding between the ADC and its target. *In vitro*, the affinity of HS630 and Kadcyla® was produced via the SPR technique on Biacore™ T200. The K_D_ values of HS630 and Kadcyla® for HER2 were 6.372 E-11M and 9.424 E-11 M, respectively. As suggested by the Biacore criteria, there is no difference in binding affinity if the difference is less than five-fold. In view of the above values, no significant difference was observed in the binding biological activities of HS630 and Kadcyla® to HER2.

HS630 is an ADC that can be metabolized into a total antibody and small molecule drug *in vivo* (total antibody: the sum of naked antibodies and ADC; ADC: antibody-coupled small molecule drug; small molecule: broken part of the small molecule drug). To accurately quantify the concentration *in vivo*, this study used double-antibody sandwich ELISA for total antibody and ADC, as well as HPLC-MS/MS for DM1. Double-antibody sandwich ELISA was used to determine the concentration of total antibody (including naked antibody and ADC) and the ADC of HS630 and Kadcyla® in the serum and tumors of tumor-bearing mice (Damen et al., 2009; Lee et al., 2011; Kaur et al., 2013; Kozak et al., 2013). When detecting total antibody concentration, the coated primary antibody was HER2, which can simultaneously bind naked antibody and ADC. Meanwhile, the coated primary antibody for detecting the ADC was the specific anti-small molecule drug antibody, which can only detect the ADC in the sample. The goat anti-human mAb adsorbed by HRP-labeled monkey serum was utilized to detect the Fc fragment of the binding antibody of the secondary antibody (Dere et al., 2013; Barok et al., 2014). Based on the results of method optimization and validation, a series of *in vivo* pharmacokinetic experiments were carried out to verify the similarity.

*In vivo*, total antibody concentration (including naked antibody and ADC) in the serum of HS630 and Kadcyla® groups peaked at 5 min and then gradually decreased after 10 mg·kg^−1^ HS630 and Kadcyla® were injected into the tail vein of tumor-bearing mice. WinNonlin was used for biosimilarity evaluation. According to the guidelines of FDA, NMPA, and WHO for pharmacokinetic similarity assessment, the 90% CI of the primary endpoint for pharmacokinetic studies such as C_max_ and AUC_(0–t)_ should fall within the range of 80%–125% on the evaluation of biosimilars (FDA., 2014; NMPA., 2021; WHO., 2022). The 90% CI of the C_max_ geometric mean ratio of HS630 and reference preparation Kadcyla® was 89.205%, and the 90% CI of AUC_(0–t)_ geometric mean ratio was 88.186%, both of which met the aforementioned evaluation criteria. The ADC concentration in the serum of HS630 and Kadcyla® groups peaked at 5 min as total antibody and then gradually decreased. The 90% CI of the C_max_ geometric mean ratio of HS630 and reference preparation Kadcyla® was 89.270% and the 90% CI of the AUC_(0–t)_ geometric mean ratio was 80.169%, both of which fell within the standard range (80%–125%). Furthermore, in order to evaluate the post-injection tissue distribution of HS630 and Kadcyla® into tumor, this study also measured the drug concentrations in tumors at various time points after administration. After tail vein injection of 10 mg·kg^−1^ HS630 and Kadcyla® into tumor-bearing mice, the total antibody concentration (including naked antibody and ADC) in the tumor peaked at 24 and then gradually decreased. With the WinNonlin used for biosimilarity evaluation, the 90% CI of the geometric mean ratio of C_max_ for HS630 and the control was 128.036%, and the 90% CI of the geometric mean ratio of AUC_(0–t)_ was 101.185%, meeting the above criteria. The conjugated drug (ADC) in tumor tissue could not be detected using ELISA. It can be inferred that the ADC in the tumor tissue antibody to the small molecule drug (DM1) was basically broken, and the drug played a role after entering the tumor tissue (Erickson et al., 2006; Kaur et al., 2013; Barok et al., 2014). The sample size required for ELISA is large, and the sample processing of ELISA is different from that of HPLC-MS/MS. In our study, there was a small volume of tumor-bearing mice and an insufficient amount of serum and tumor samples that could be provided. Thus, the small molecule drugs in the serum and tumor tissue of tumor-bearing mice are limited by the number of samples not examined, which should be verified in subsequent testing of experimental samples for toxicities and drug delivery.

In general, nonhuman primates (NHPs) such as cynomolgus monkeys, given their high consistency with humans in target homology, metabolic pathways, and drug responses, are used for nonclinical pharmacokinetic and toxicological studies of ADC. The efficacy of an ADC depends on their specific binding to target antigens. Significantly, the target proteins of NHPs exhibit high homology to those of humans, supporting their abilities to stimulate binding efficiency and specificity of the drug in humans. Meanwhile, NHPs share high similarities in the hepatic metabolic enzymes and clearance mechanisms to humans, allowing for the accurate prediction of their absorption, distribution, metabolism, and excretion in humans. Therefore, in this study, a series of pharmacokinetic experiments were conducted to evaluate the similarity between HS630 and Kadcyla®.

Following a single intravenous infusion of HS630 at 0.33 mg·kg^−1^, 1 mg·kg^−1^, and 3 mg·kg^−1^ in cynomolgus monkeys, the serum concentration of the ADC increased with the dose administered. The peak times were all at 0.5 h after administration, followed by a slower elimination, and the serum-drug concentrations were detectable up to 504 h for all three dose groups. The ratio of doses, AUC_(0–504 h)_, and C_max_ was 1:3:9, 1:6.67:24.23, and 1:5.23:12.69, respectively, suggesting that within the dose range of 0.33∼3 mg·kg^−1^, HS630 behaves as a non-linear pharmacokinetic profile in cynomolgus monkeys. Meanwhile, through continuous intravenous infusions of 3 mg·kg^−1^ HS630 in cynomolgus monkeys, T_max_ was at 0.5 h after the first dose and every 504 h thereafter. The serum concentration of the ADC showed an obvious peak–trough trend with the number of administrations and peaked 0.5 h after the last (fourth) dose, followed by a slower elimination, with the serum drug concentration detectable up to 1,008 h after the last dose. There was a difference between the last and first doses at 0 and 504 h, yet without any significant difference in any of the remaining time points. AUC_(0–504 h)_ and C_max_ after the last dose were not statistically significantly different from after the first dose, and the accumulation factor was 1.04 ± 0.20, revealing no significant drug accumulation produced after the continuous administration of HS630 at 3 mg·kg^−1^.

After a single intravenous infusion of Kadcyla® at 3 mg·kg^−1^ in cynomolgus monkeys, T_max_ was also 0.5 h after administration, followed by a slower elimination, and the serum-drug concentration of the ADC was detectable up to 504 h, without statistically significant differences between HS630 and the control at all time points except for 72 and 504 h. WinNonlin was used for biosimilarity evaluation, and the 90% CI of the ratio of geometric means of C_max_ for HS630 and Kadcyla® was 109.15%, meeting the criteria for biosimilarity of 80%–125%. Similarly, the 90% CI for the geometric mean ratio of AUC_(0–t)_ was 97.51%, also meeting the biosimilarity decision criteria of 80%–125%.

Furthermore, the total antibody concentration increased with the administration dose according to the results observed, following a single intravenous infusion of HS630 at 0.33 mg·kg^−1^, 1 mg·kg^−1^, and 3 mg·kg^−1^ in cynomolgus monkeys. The peak times were all at 0.5 h after administration, followed by a slower elimination, and the serum drug concentrations were detectable up to 504 h for all three dose groups. The ratios of dosage, AUC_(0–504 h)_, and C_max_ were 1:3:9, 1:6.46: 29.08, and 1:5.12: 16.90, respectively. Therefore, the total antibody in 0.33∼3 mg·kg^−1^ of HS630 exhibited non-linear pharmacokinetic characteristics in cynomolgus monkeys.

With continuous intravenous infusions of 3 mg·kg^−1^ HS630 in cynomolgus monkeys, total antibody T_max_ was at 0.5 h after the first dose, followed by every 504-h administration. The total antibody concentration showed an obvious peak–trough trend with the number of administrations, and the serum concentration peaked at 0.5 h after the last (fourth) dose followed by a slower elimination, with the serum drug concentration detectable up to 1,008 h after the last dose. There was a difference between the last and first dose at 0 and 504 h but without significant difference in any of the remaining time points. However, no statistically significant difference was observed in AUC_(0–504 h)_ and C_max_ after the last dose and the first dose, and the accumulation factor was 1.22 ± 0.18, suggesting no significant drug accumulation with continuous administration of HS630 3 mg·kg^−1^.

After a single intravenous infusion of Kadcyla® at 3 mg·kg^−1^ in cynomolgus monkeys, the T_max_ of total antibody was also 0.5 h after administration, followed by a slower elimination with detectable serum drug concentrations to 504 h after the administration of HS630 and Kadcyla® at each time point. There were statistically significant differences in the total antibody serum concentrations at 24, 48, 96, 120, 168, and 504 h but without any statistical difference in other time points. WinNonlin was used for biosimilarity assessment. The 90% CI of the geometric mean ratio of the total antibody C_max_ for HS630 and Kadcyla® was 116.23%, which met the bioequivalent decision criteria of 80%–125%; that of AUC_(0–t)_ was 136.16%, which basically met the criteria. No anti-drug antibody was detected in the serum samples from cynomolgus monkeys after intravenous administration of HS630 and Kadcyla®. However, the possibility of a fortuitous result cannot be excluded, considering the limited number of administrations and the small sample size of animals in this study. We recommend paying close attention to clinical trials, given the individual differences in animal experiments.

Altogether, this study designed and conducted *in vivo* pharmacokinetic experiments to comprehensively characterize the pharmacokinetic profile of HS630 and validate the similarity to the originator Kadcyla®. These experiments evaluated dose dependency and compared the dose effects of continuous and direct similarity of HS630 in cynomolgus monkeys. The similarity between HS630 and Kadcyla® was assessed by analyzing their pharmacokinetic changes in cynomolgus monkeys with single and multiple administrations. The post-administration concentration of total antibody and ADC remained essentially consistent, with the observation of relative similarity in multiple pharmacokinetic parameters. Combined with the pharmacokinetic results of tumor-bearing mice and pharmacokinetic guidelines, HS630 exhibited a high degree of similarity of Kadcyla®. It should be acknowledged that pharmacokinetic experiments provided critical data to support the similarity of HS630 and Kadcyla®. However, this study still has several limitations, which should be considered when interpreting the findings and extrapolating these to clinical settings. In the tissue distribution experiment of tumor-bearing mice, this study failed to investigate the distribution in other organs, despite the detection of a concentration in tumors. There is a need to determine drug concentrations in more tissues to fully illustrate the safety and potential unintended off-target effects. Meanwhile, the pharmacokinetic experiments were conducted in a relatively small number of cynomolgus monkeys. Although it was sufficient to detect major pharmacokinetic differences between HS630 and Kadcyla®, there might be a lack of statistical power to assess subtle individual variability or sex-specific pharmacokinetic differences. Moreover, the pharmacokinetic profiles of HS630 and Kadcyla® exhibited similarities, although these were insufficient to fully confirm their pharmacological consistency. Our *in vitro* studies evaluated the binding ability of HS630 and Kadcyla® to HER2 to assess their protein affinity and potential therapeutic efficacy. However, the pharmacodynamic indicators need to be reflected through the actual therapeutic effects of model animals in nonclinical trials, highlighting the necessity for additional *in vivo* animal studies to provide further supportive evidence in the future.

## 5 Conclusion

The present study developed a method of ELISA for detecting total antibody and ADC drugs of HS630. Its specificity, precision, and accuracy in measuring total antibody and ADC fulfill the requirements of pharmacokinetic studies. HS630 and the originator drug Kadcyla® revealed pharmacokinetic similarity after intravenous infusion in tumor-bearing mice and cynomolgus monkeys. Comprehensive nonclinical evaluations of this study provide robust evidence for regulatory approval, beyond addressing key scientific and technical challenges in biosimilar development. Eventually, the findings of this study may contribute to the acceleration of the translation of HS630 and related biosimilars from laboratory to the market, as well as improving patients’ opportunities to obtain high-quality, cost-efficient biotherapeutics.

## Data Availability

The original contributions presented in the study are included in the article/Supplementary Material; further inquiries can be directed to the corresponding author.
