# Supplementary material for: Nonclinical evaluation of HS630, a proposed biosimilar of trastuzumab emtansine: affinity, pharmacokinetics, and immunogenicity
Source: Front Pharmacol. 2025 Dec 18;16:1698727. doi: 10.3389/fphar.2025.1698727 (PMC12756435; doi:10.3389/fphar.2025.1698727)
Supplement: Supplementary file 1 [file Supplementaryfile1.docx]

Appendix I HS630 Enzyme linked immunosorbent assay (ELISA) assay development and validation

# Materials and Reagents

HS630, provided by Zhejiang Hisun Pharmaceutical Co., Ltd. Batch number: 20131101, specification: 100 mg, powder, stored at 2~8 ℃ in the dark. Control drug: Kadcyla^®^. Manufactured by Roche and provided by Zhejiang Hisun Pharmaceutical Co., Ltd. lot No: 566313 at a concentration of 100 mg/vial as a powder and stored at 2~8 ℃ in the dark. Anti-DM1 antibody, provided by Zhejiang Hisun Pharmaceutical Co., Ltd.), hGoat anti-Human IgG-heavy and light chain monkey-adsorbed Antibody HRP Conjugated (Bethyl Laboratories, Inc.,), Skim Milk (BD Difco^TM^), BSA (F. Hoffmann-La Roche&Co. Ltd ), TMB single component color development solution (Beijing Solarbio Science & Technology Co.,Ltd.), H_2_SO_4_ (Sinopharm Chemical Reagent Co., Ltd.), NaCl (xilonghua Co., Ltd.), KCl (Sinopharm Chemical Reagent Co., Ltd.), KH_2_PO_4_ (Sinopharm Chemical Reagent Co., Ltd.), Na_2_HPO_4_ (Sinopharm Chemical Reagent Co., Ltd.), Tween 20 (Sinopharm Chemical Reagent Co., Ltd.).

# Method validation and results

## Standard curve and linear range

HS630 was diluted with blank cynomolgus monkey serum at 20% concentration into standards of 100, 50, 25, 12.5, 6.25, 3.125, and 1.5625 ng·mL^-1^, 100 µL of each standard was taken and detected with the assay described above. The OD 450 nm-560 nm was used as the ordinate and the concentration of each standard was used as the abscissa, which was subjected to non linear regression operation, and the obtained regression equation was the standard curve of HS630. The fitted curves are presented in Supplementary Table 1 and Supplementary Figure 1. Calibration curves were fitted by a four parameter logistic function model, and the slope, EC50, and near linear range between the upper and lower asymptotes were evaluated. Its mathematical model is:

$$\boldsymbol{Y}\boldsymbol{=}\frac{\boldsymbol{(A}\boldsymbol{1-A}\boldsymbol{2)}}{\boldsymbol{1+}{\boldsymbol{(X}\boldsymbol{/}\boldsymbol{X}\boldsymbol{0)}}^{\boldsymbol{p}}}\boldsymbol{+}\boldsymbol{A}\boldsymbol{2}$$

Where A1 is the estimate of the lower end asymptotes of the sigmoidal curve, and A2 is the estimate of the upper end asymptotes of the sigmoidal curve. For samples which content out of the near linear range between the upper and lower asymptotes, dilute appropriately with diluent to the calibration curve optimal measured concentration range for the assay. Unknown serum samples concentration were calculated with their respective standard calibration curves on the same plate.

Supplementary Table 1 Standard curve and linearity range

| **Expected（ng·mL^-1^）** | **OD1** | **OD2** | **Mean** | **Mean-KB** | **SD** |
| --- | --- | --- | --- | --- | --- |
| 1.5625 | 0.2022 | 0.2038 | 0.2030 | 0.124 | 0.001 |
| 3.125 | 0.3135 | 0.3169 | 0.3152 | 0.236 | 0.002 |
| 6.25 | 0.4671 | 0.4974 | 0.4823 | 0.403 | 0.021 |
| 12.5 | 0.7479 | 0.7614 | 0.7547 | 0.676 | 0.010 |
| 25 | 1.2273 | 1.2306 | 1.2290 | 1.150 | 0.002 |
| 50 | 1.9236 | 2.0140 | 1.9688 | 1.890 | 0.064 |
| 100 | 2.7208 | 2.7656 | 2.7432 | 2.664 | 0.032 |
| KB | 0.0825 | 0.0757 | 0.0791 | 0 | 0.005 |

Note: KB for blank wells.


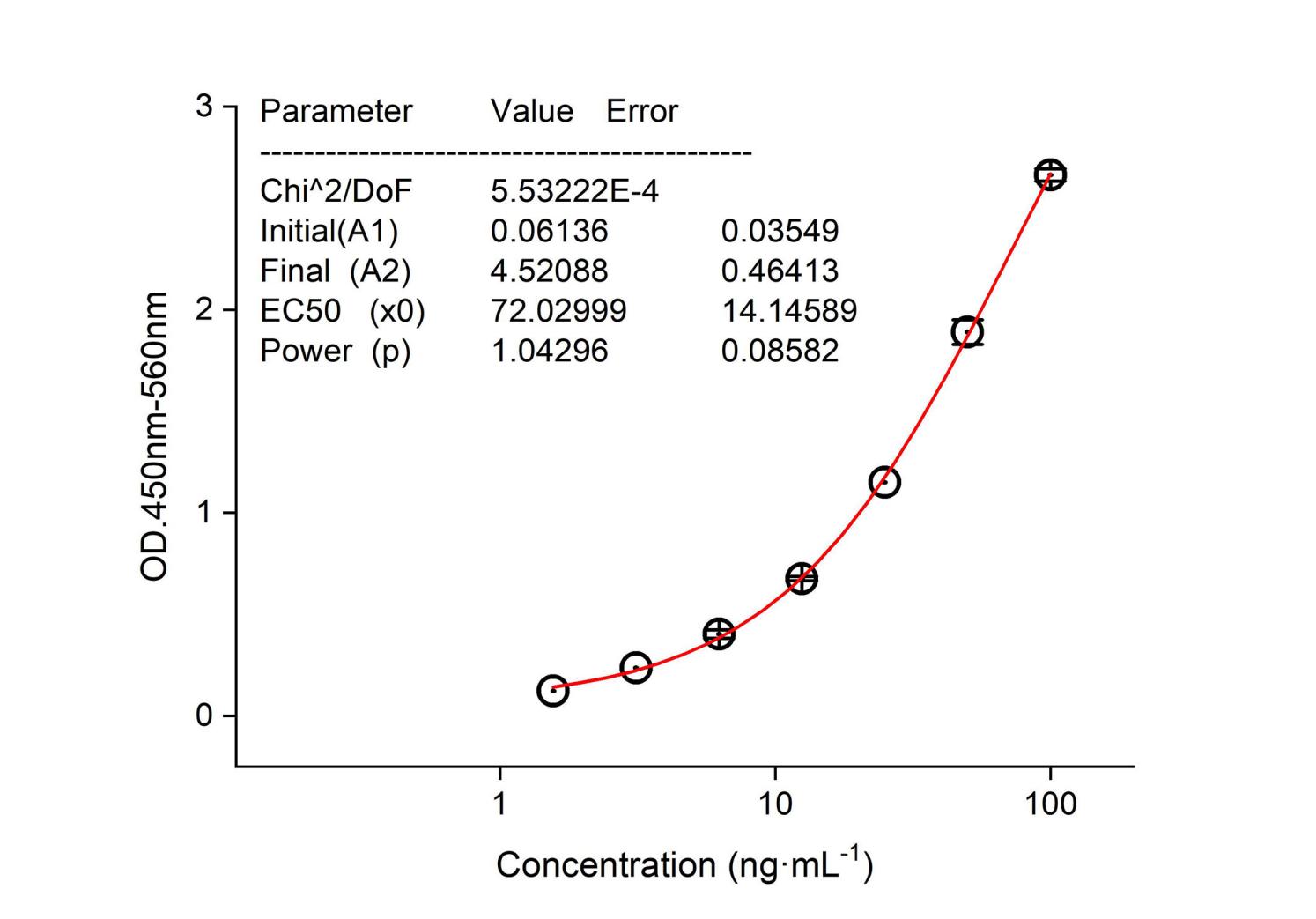


**Supplementary Figure 1.** Four-Parameter Logistic calibration curve of HS630

## Sensitivity and limit of quantitationof detection (LOQ)

The test article HS630 at concentration of 1.5625 ng·mL^-1^ was made according to the method for preparing the standard curve, the assay was performed similarly as described above, and repeated eight times. The concentration calculated from the standard curve regression equation on the same plate was the detected amount of HS630. The standard recovery test met the LOQ of 1.5625 ng·mL^-1^ with intra assay CV% < 20% (4.47%).

Supplementary Table 2 Sensitivity of HS630 by ELISA methods with spiked in cynomolgus monkey serum

| **Expected（ng·mL^-1^）** | **Measured values（ng·mL^-1^）** | **RE(%)** | **Mean RE(%)** | **CV(%)** |
| --- | --- | --- | --- | --- |
| 1.5625 | 1.5770 | 0.93 | +9.07 | 4.47 |
|  | 1.8003 | 15.22 |  |  |
|  | 1.7985 | 15.10 |  |  |
|  | 1.6622 | 6.38 |  |  |
|  | 1.6584 | 6.14 |  |  |
|  | 1.7511 | 12.07 |  |  |
|  | 1.6887 | 8.07 |  |  |
|  | 1.6981 | 8.68 |  |  |

## Precision and accuracy

The assay was performed similarly as described above for measured high, medium and low concentrations of 80, 16, 2 ng·mL^-1^, and each concentration was assayed 8 times in parallel for a total of 3 times to calculate the intra assay CV, inter assay CV values and the RE values respectively. The results showed that the intra and inter assay CVS at the three concentration levels, high, medium and low, were less than 15%, indicated the detection method showed high precision; At the same time, the re values between the intra and inter assay at the high, medium and low concentration levels were less than 15%, indicated the detection method showed high accuracy.

Supplementary Table 3 Precision and accuracy detection of HS630 by ELISA methods with spiked in cynomolgus monkey serum

|  | **Expected (ng·mL^-1^)** | **Measured (ng·mL^-1^)** | **CV%** | **RE%** |
| --- | --- | --- | --- | --- |
| **Intra assay（n=8）** | 2 | 2.02±0.16 | 7.77 | +1.08 |
|  | 16 | 16.59±0.95 | 5.74 | +3.67 |
|  | 80 | 81.7±5.85 | 7.16 | +2.13 |
| **Inter assay（n=24）** | 2 | 1.99±0.16 | 8.06 | -3.61 |
|  | 16 | 16.56±0.89 | 5.40 | +1.66 |
|  | 80 | 83.35±7.24 | 8.68 | -5.88 |

## Specificity

HS630 was made into 100 ng·mL^-1^ - 1.5625 ng·mL^-1^ as the method for preparing the standard curve described above, while the same concentration gradient samples were prepared for Kadcyla^®^, Trastuzumab (Herceptin), Bevacizumab, Adalimumab and Infliximab, and the detection results are shown in Supplementary Figure 2. HS630 showed no cross reactivity with Trastuzumab (Herceptin) , Bevacizumab,Adalimumab and Infliximab, and good methodological specificity. The standard curve of HS630 and control drug Kadcyla^®^ show high degree of coincidence, indicated consistency of the detection method is good for detection.


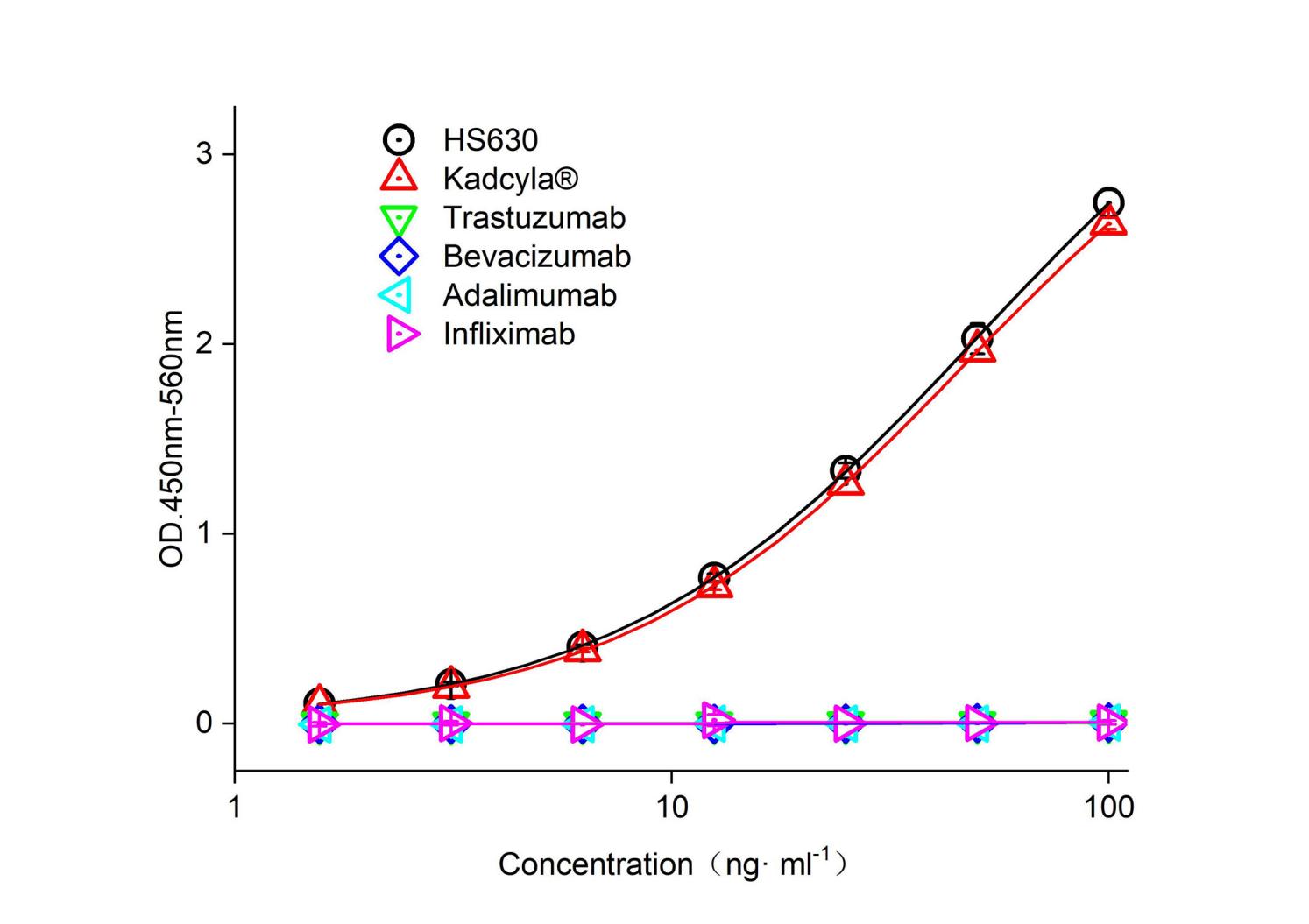


**Supplementary Figure 2.**  Specificity validation curve of HS630

## Matrix effect

The standard curves were prepared with 100% cynomolgus monkey serum, tumor diluent, 20% cynomolgus monkey serum, 50% cynomolgus monkey serum, 20% SD rat serum and 20% tumor bearing mice serum as diluents respectively. The results are shown in Supplementary Figure 3. The standard curves of 20% cynomolgus monkey serum, 20% SD rat serum and 20% tumor bearing mice serum were comparatively coincident. Therefore, considering the limited blood volume of tumor-bearing mice in the pharmacodynamic experiment, 20% serum of SD rats was used as the diluent for the serum samples of tumor bearing mice.


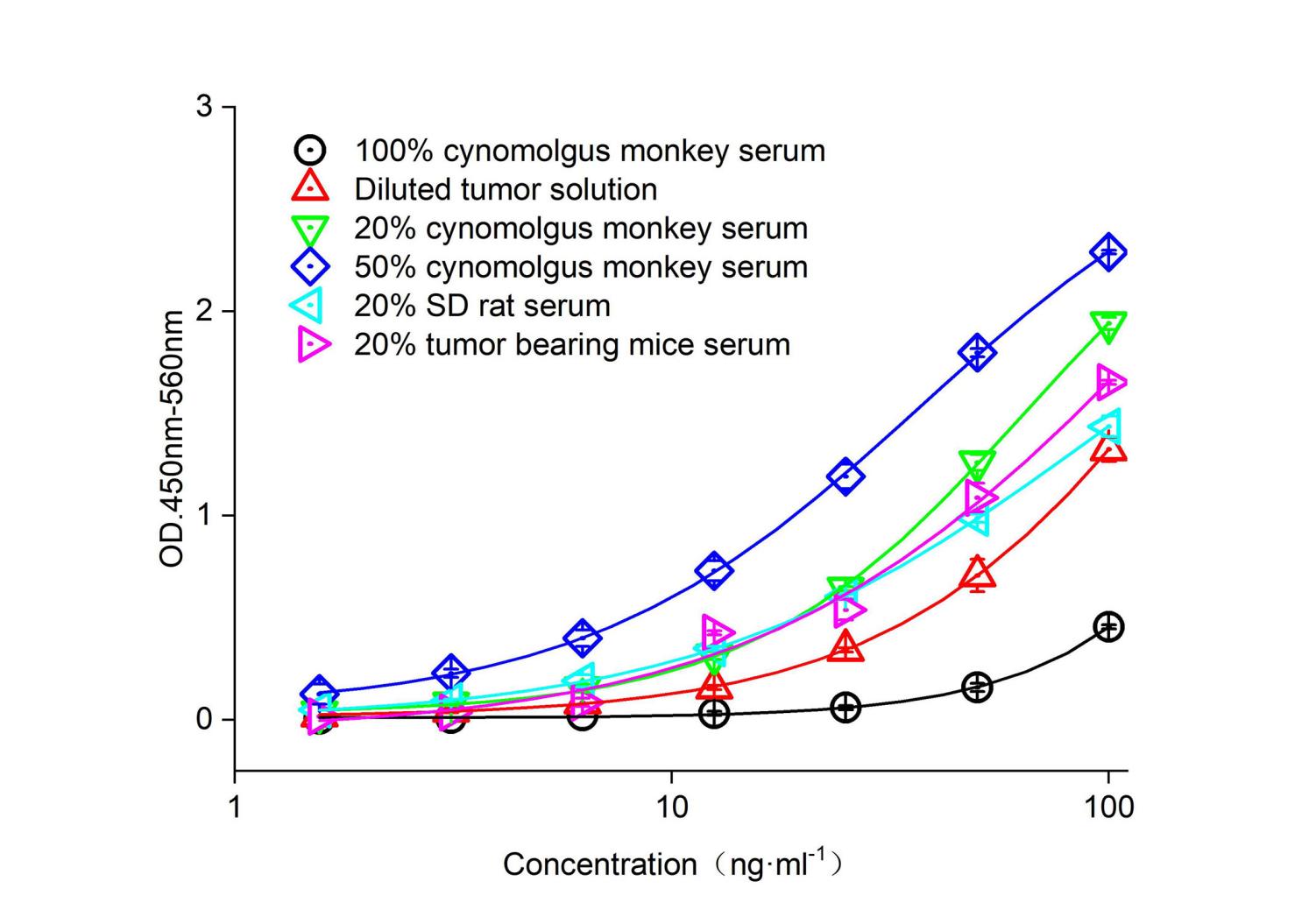


**Supplementary Figure 3.**  Matrix effect comparison curve

## Stability

The results of the stability study of HS630 in monkey serum are shown in Supplementary Table 4. The results show that the stability of HS630 in serum at -80 ℃ (after three freeze/thaw cycles), at room temperature for 4 h, at 4 ℃ for 4 h, and at - 80 ℃ for 40 and 100 days did not affect its stability, but long-term storage at -80 ℃ for 160 days did not guarantee its stability, therefore, testing of samples was completed before 160 days.

Supplementary Table 4-1 Stability of HS630 (unit: ng·mL^-1^)

| **Treatment conditions** | **Freeze thaw 3 times** | | |  | **Room temperature 4 h** | | |  | **4℃ 4h** | | |  |
| --- | --- | --- | --- | --- | --- | --- | --- | --- | --- | --- | --- | --- |
| **Expected** | 2 | 16 | 80 |  | 2 | 16 | 80 |  | 2 | 16 | 80 |  |
| **Measured values** | 1.98 | 17.53 | 69.84 |  | 2.02 | 17.52 | 71.25 |  | 1.99 | 17.56 | 70.91 |  |
|  | 2.11 | 17.20 | 93.12 |  | 2.13 | 17.19 | 90.71 |  | 2.10 | 17.20 | 91.57 |  |
|  | 1.79 | 17.63 | 88.03 |  | 1.81 | 17.62 | 86.42 |  | 1.80 | 17.61 | 86.98 |  |
|  | 1.75 | 16.21 | 98.07 |  | 1.78 | 16.19 | 93.86 |  | 1.74 | 16.19 | 95.39 |  |
|  | 1.72 | 14.97 | 101.38 |  | 1.75 | 14.97 | 96.94 |  | 1.73 | 14.95 | 98.95 |  |
|  | 2.15 | 15.75 | 84.81 |  | 2.15 | 15.74 | 82.92 |  | 2.14 | 15.75 | 83.67 |  |
|  | 2.11 | 16.57 | 72.64 |  | 2.12 | 16.55 | 72.02 |  | 2.11 | 16.56 | 72.36 |  |
|  | 2.04 | 16.23 | 91.97 |  | 2.06 | 16.24 | 90.24 |  | 2.05 | 16.25 | 91.42 |  |
| **Mean** | 1.96 | 16.51 | 87.48 |  | 1.98 | 16.50 | 85.55 |  | 1.96 | 16.51 | 86.40 |  |
| **RE(%)** | -2.19 | +3.19 | +9.36 |  | -1.16 | +3.14 | +6.93 |  | -2.03 | +3.19 | +8.01 |  |
| **SD** | 0.18 | 0.92 | 11.31 |  | 0.17 | 0.92 | 9.58 |  | 0.18 | 0.92 | 10.25 |  |
| **CV(%)** | 9.00 | 5.57 | 12.93 |  | 8.53 | 5.55 | 11.20 |  | 8.95 | 5.60 | 11.86 |  |

Supplementary Table 4-2 HS630 stability (unit: ng·mL^-1^)

| **Treatment conditions** | **Stored at -80 °C for 40 days** | | |  | **Stored at -80 °C for 100 days** | | |  | **Stored at -80 °C for 160 days** | | |  |
| --- | --- | --- | --- | --- | --- | --- | --- | --- | --- | --- | --- | --- |
| **Expected** | 2 | 16 | 80 |  | 2 | 16 | 80 |  | 2 | 16 | 80 |  |
| **Measured values** | 1.96 | 17.48 | 67.54 |  | 1.96 | 17.43 | 66.52 |  | 1.96 | 17.37 | 65.00 |  |
|  | 2.09 | 17.15 | 97.60 |  | 2.09 | 17.10 | 99.74 |  | 2.10 | 17.06 | 107.77 |  |
|  | 1.76 | 17.57 | 91.85 |  | 1.77 | 17.56 | 93.83 |  | 1.76 | 17.52 | 102.50 |  |
|  | 1.73 | 16.15 | 106.36 |  | 1.73 | 16.12 | 112.10 |  | 1.72 | 16.05 | 134.89 |  |
|  | 1.69 | 14.93 | 110.16 |  | 1.69 | 14.91 | 117.57 |  | 1.70 | 14.85 | 143.01 |  |
|  | 2.13 | 15.70 | 88.49 |  | 2.14 | 15.67 | 91.19 |  | 2.14 | 15.61 | 98.26 |  |
|  | 2.10 | 16.54 | 73.90 |  | 2.10 | 16.51 | 74.71 |  | 2.10 | 16.44 | 77.90 |  |
|  | 2.02 | 16.18 | 95.46 |  | 2.03 | 16.16 | 97.91 |  | 2.03 | 16.08 | 104.29 |  |
| **Mean** | 1.94 | 16.46 | 91.42 |  | 1.94 | 16.43 | 94.20 |  | 1.94 | 16.37 | 104.20 |  |
| **RE(%)** | -3.22 | 2.89 | 14.28 |  | -3.16 | 2.71 | 17.75 |  | -2.96 | 2.33 | 30.25 |  |
| **SD** | 0.18 | 0.91 | 14.71 |  | 0.18 | 0.91 | 17.18 |  | 0.19 | 0.91 | 25.99 |  |
| **CV(%)** | 9.35 | 5.55 | 16.09 |  | 9.39 | 5.54 | 18.24 |  | 9.56 | 5.59 | 24.94 |  |

## Effect of sample dilution factor

HS630 was prepared with 20% monkey serum at a concentration of 1mg·mL^-1^, then diluted it in 12500 fold, 62500 fold, and 500000 fold with 8 times in parallel for detected. The results are shown in Supplementary Table 5. Indicating that diluting HS630 standard solution with 20% monkey serum at 12500 fold, 62500 fold, and 500000 fold had no effect on sample detection and there is no dilution effect.

Supplementary Table 5 Effect of sample dilution factor

|  | **500000 fold dilution** | **62500 fold dilution** | **12500 fold dilution** |
| --- | --- | --- | --- |
| **Measured values**  **ng·mL^-1^** | 1.97 | 17.50 | 68.62 |
|  | 2.10 | 17.17 | 94.11 |
|  | 1.78 | 17.59 | 88.73 |
|  | 1.74 | 16.17 | 99.77 |
|  | 1.71 | 14.95 | 104.04 |
|  | 2.13 | 15.72 | 86.08 |
|  | 2.10 | 16.54 | 72.75 |
|  | 2.02 | 16.20 | 93.26 |
| **Mean(ng·mL^-1^)** | 1.94 | 16.48 | 88.42 |
| **RE(%)** | -2.76 | +3.01 | +10.52 |
| **SD** | 0.18 | 0.91 | 12.37 |
| **CV(%)** | 9.02 | 5.55 | 14.00 |

## Recovery

The results of HS630 recovery from monkey serum are shown in Supplementary Table 6, with CV%<15% (9.02%, +5.55%, +14.00%) and RE%<15% (-2.76%, +3.01%, +10.52%), the recoveries met the methodological requirements.

Supplementary Table 6 methodological recoveries

| **Expected（ng·mL^-1^）** | **Measured (ng·mL^-1^)** | **CV%** | **RE%** |
| --- | --- | --- | --- |
| 2 | 1.94±0.18 | 9.02 | -2.76 |
| 16 | 16.48±0.91 | 5.55 | +3.01 |
| 80 | 88.42±12.37 | 14.00 | +10.52 |

## Summary

The linear range of HS630 ELISA is 1.5625 to 100 ng·mL^-1^, the recovery of LOQ (1.5625 ng·mL^-1^) was evaluateed as intra assay CV%<20% (4.47%). The intra assay precision was 7.77%, 5.74%, and 7.16% for the high, medium, and low concentrations, respectively, the intra assay accuracy was +1.08%, +3.67%, and +2.13%. The inter assay precision was 8.06%, 5.40% and 8.68%, and the inter assay accuracy was -3.61%, +1.66% and -5.88% for the high, medium and low concentrations, respectively. This method had excellent specificity, and there is no cross reactivity of Trastuzumab, Bevacizumab, Adalimumab or Infliximab. HS630 stored in serum at - 80 ℃ (after three freeze/thaw cycles), at room temperature for 4 h, at 4 ℃ for 4 h, and at -80 ℃ for 40 and 100 days did not affect its stability, but long-term storage at -80 ℃ for 160 days did not guarantee its stability, therefore, testing of samples was completed before 160 days. And no effect on sample detection after 12500 fold, 62500 fold, and 500000 fold dilutions were performed. The methodological validation showed that the specificity, precision and accuracy of the ELISA method for measuring HS630 concentrations in monkey serum fulfilled the requirements of pharmacokinetic studies.
