# Supplementary material for: Nonclinical evaluation of HS630, a proposed biosimilar of trastuzumab emtansine: affinity, pharmacokinetics, and immunogenicity
Source: Front Pharmacol. 2025 Dec 18;16:1698727. doi: 10.3389/fphar.2025.1698727 (PMC12756435; doi:10.3389/fphar.2025.1698727)
Supplement: Supplementary file 2 [file Supplementaryfile3.docx]

Appendix III Establishment and validation of DM1 detection by HPLC-MS / MS

# Materials and Reagents

Standard: DM1, manufactured by Zhejiang Hisun Pharmaceutical Co., Ltd., lot: s140101;Internal standard (IS): maytansinol, manufactured by Zhejiang Hisun Pharmaceutical Co., Ltd., lot No.: s131102; Acetonitrile, chromatographically pure, manufactured by Fisher Scientific, lot: 135525; Methanol, chromatographically pure, manufactured by Fisher Scientific, lot: 134191; Reducing agent TCEP, Thermo Scientific, lot: qc212998; Formic acid, chromatography pure, Honeywell International Inc. lot: hx090818; Ammonium formate, analytical grade, Sinopharm Chemical Reagent Co., Ltd., lot: 20140115; Water, milipore ultrapure water.

# Method validation and results

## HPLC-MS / MS conditions

High performance liquid chromatography (HPLC): Spark Symbiosis system; Chromatographic column: Aglient Eclipse C18(100mm×2.1mm, 3.5μm); Column temperature: 20 ℃; Flow rate: 0.3 mL/min; Auto-sampler temperature: 4 ℃; Injection volume: 10μL; Mobile phases: organic phase (B), maytanol; aqueous phase (A), deionized water (consisted of 8mM ammonium formate and 0.1% formic acid); Gradient elution: 0–1.0 min 10% B→ 90% B; 1.0–2.5 min 90% B→ 90% B; 2.5–4.03 min 90% B→ 10% B; 4.06–6.0 min 10% B→ 10% B. Mass spectrometry: AB API 4000QTrap; Ion source: ESI source; Ion mode: positive; Drying gas (N2):50 L·mL^-1^; Drying gas temperature: 500 ℃; Capillary voltage: 5500V; Scan mode: MRM (Multiple Reaction Monitoring); Ion pairs: m/z 738.54→547.2 (DM1), m/z 565.3→453.0(IS); Declustering Potential: 35V (DM1), 28V (IS); Collision energy: 120V (DM1), 30V (IS).

## Sample preparation

### Serum sample preparation

A 90 μL aliquot of cynomolgus monkey serum sample was mixed with 10μL TCEP, 10μL maytanol, 10μL internal standard solution (250ng·mL^-1^) and 200μL acetonitrile in the 1.5mL centrifuge tubes.The mixture was vortex mixed for 2min, and centrifuged at 14000 r·min^-1^ for 10 min at 4 °C. 10 μL of the supernatant was injected into the HPLC-MS/ MS for analysis.

### Preparation of calibration standards and quality control (QC) samples

Blank sample: A 90 μL aliquot of blank cynomolgus monkey serum sample was mixed with 10μL TCEP, 20μL maytanol and 200μL acetonitrile in the 1.5mL centrifuge tubes.The mixture was vortex mixed for 2min, and centrifuged at 14000 r·min^-1^ for 10 min at 4 ℃. 10 μL of the supernatant was injected into the HPLC-MS/ MS for analysis.

Calibration curve standard sample: A 90 μL aliquot of blank cynomolgus monkey serum sample was mixed with a different concentrations of DM1 standard solution (5.0, 15, 50, 100, 500, 1500, 2500 ng·mL^-1^) separately, to obtain the drug-containing serum at concentrations of 0.5, 1.5 ,5 , 10, 50, 150, 250 ng·mL^-1^.Then, 10μL TCEP, 10μL internal standard solution (250ng·mL^-1^) and 200μL acetonitrile were added in, the mixture was vortex mixed for 2min, and centrifuged at 14000 r·min^-1^ for 10 min at 4 ℃. 10 μL of the supernatant was injected into the HPLC-MS/ MS for analysis.

Quality control sample: A 90 μL aliquot of blank cynomolgus monkey serum sample was mixed with a different concentrations of DM1 standard solution (10, 250, 2000 ng·mL^-1^) separately, to obtain the drug-containing serum at concentrations of 1.0, 25, 200 ng·mL^-1^.Then, 10μL TCEP, 10μL internal standard solution (250ng·mL^-1^) and 200μL acetonitrile were added in, the mixture was vortex mixed for 2min, and centrifuged at 14000 r·min^-1^ for 10 min at 4 °C. 10 μL of the supernatant was injected into the HPLC-MS/ MS for analysis.

## Standard curve and linear range

The standard curve was obtained by taking the ratio of the concentration of the analyte DM1 to the internal standard as the x-axis, and the ratio of the peak area of the analyte DM1 to the internal standard as the y-axis, as shown in Supplementary Figure 1. The linear range of DM1 concentration is good within the range of 0.5-250 ng·mL^-1^, with y=0.0445x-0.00275 (R^2^=0.9991).


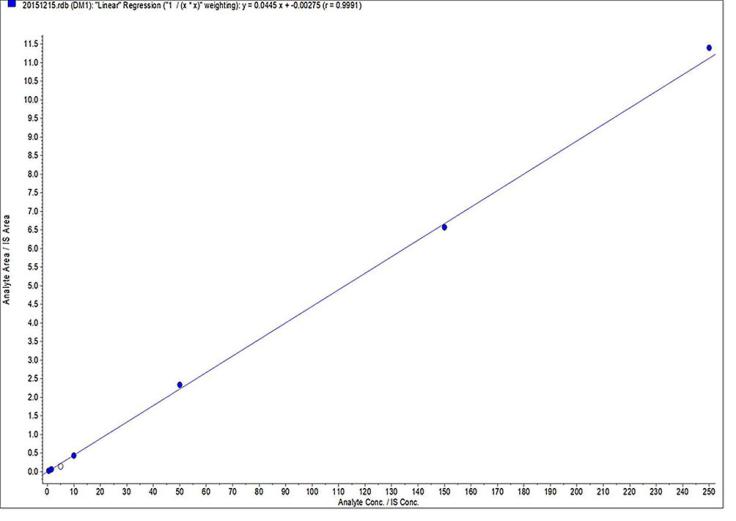


**Supplementary Figure 1.** DM1 standard curve in cynomolgus monkey serum samples

## Good specificity

As shown in Supplementary Figure 2, the standard solutions of DM1 and internal standard with concentrations of both 0.5 ng·mL^-1^ were mixed and directly injected to HPLC-MS/ MS to obtain the chromatograms of DM1 and internal standard (A); 90 μL aliquot of blank cynomolgus monkey serum followed the procedures under “2.2.1 Serum sample preparation” except for adding DM1 and internal standard, to obtain a blank serum chromatogram (B); drug-containing serum at concentrations of 0.5 ng·mL^-1^ was performanced as “2.2.1 Serum sample preparation” to obtain the chromatograms of DM1 and internal standard after extraction (C); Cynomolgus monkeys serum samples at 0.5h post dose was performanced as “2.2.1 Serum sample preparation” to obtain the chromatograms of DM1 and internal standard in the serum samples (D).


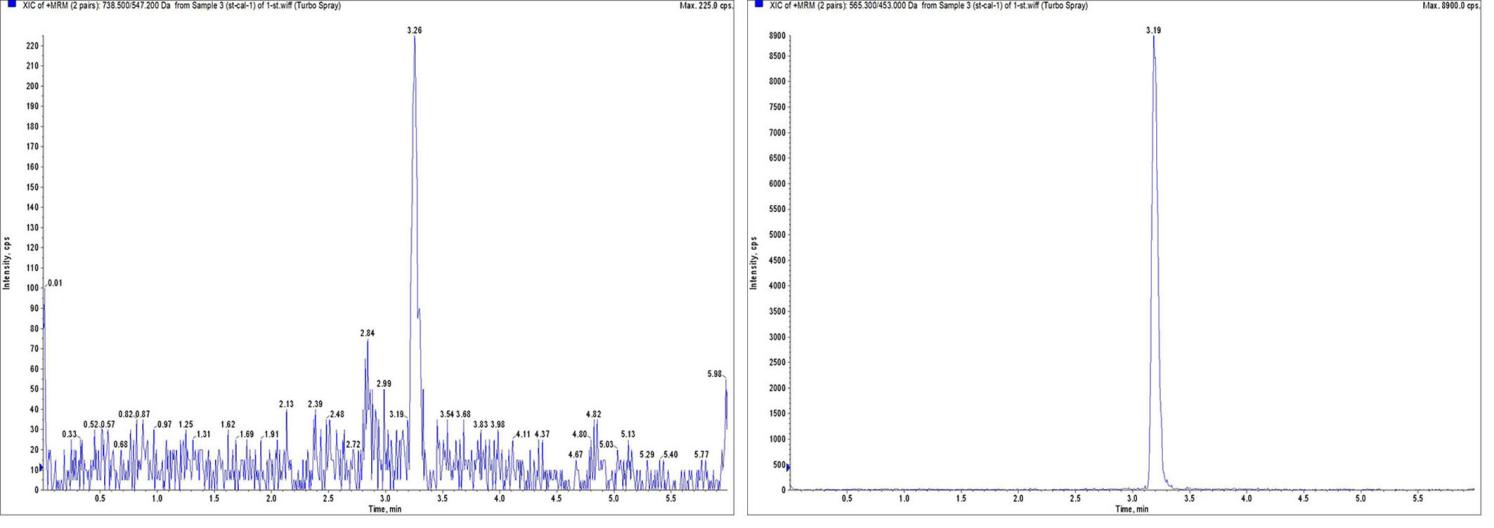


**Supplementary Figure 2A.** Chromatogram of 0.5 ng·mL^-1^ DM1 and internal standard.


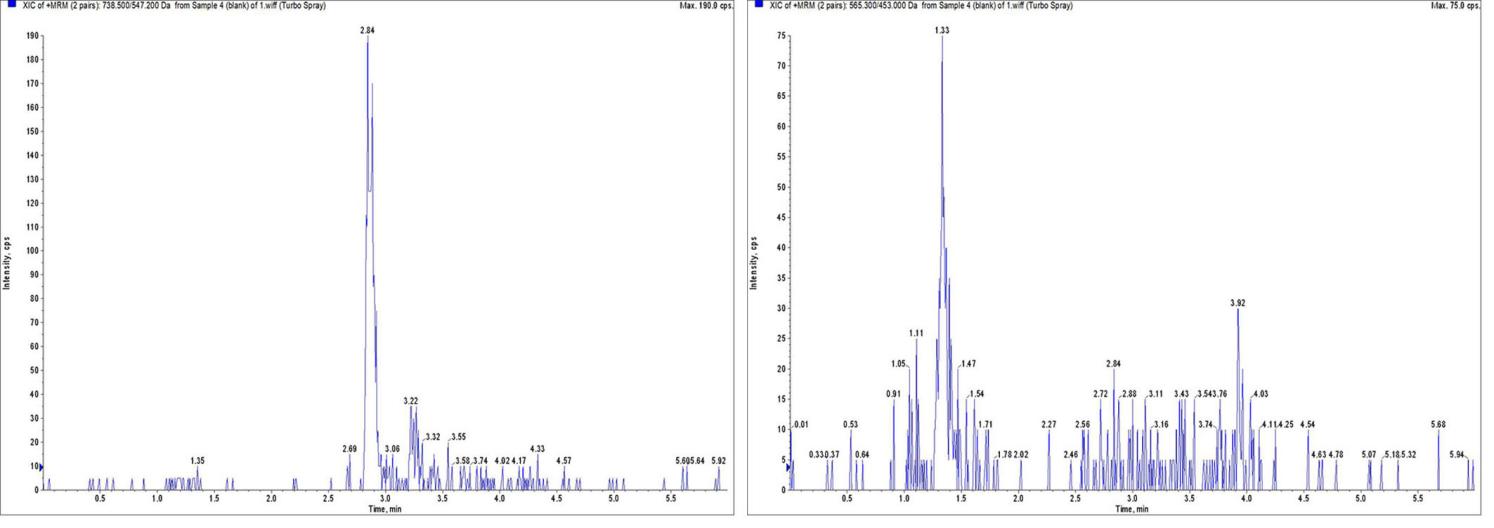


**Supplementary Figure 2B.** Chromatogram of blank cynomolgus monkey serum.


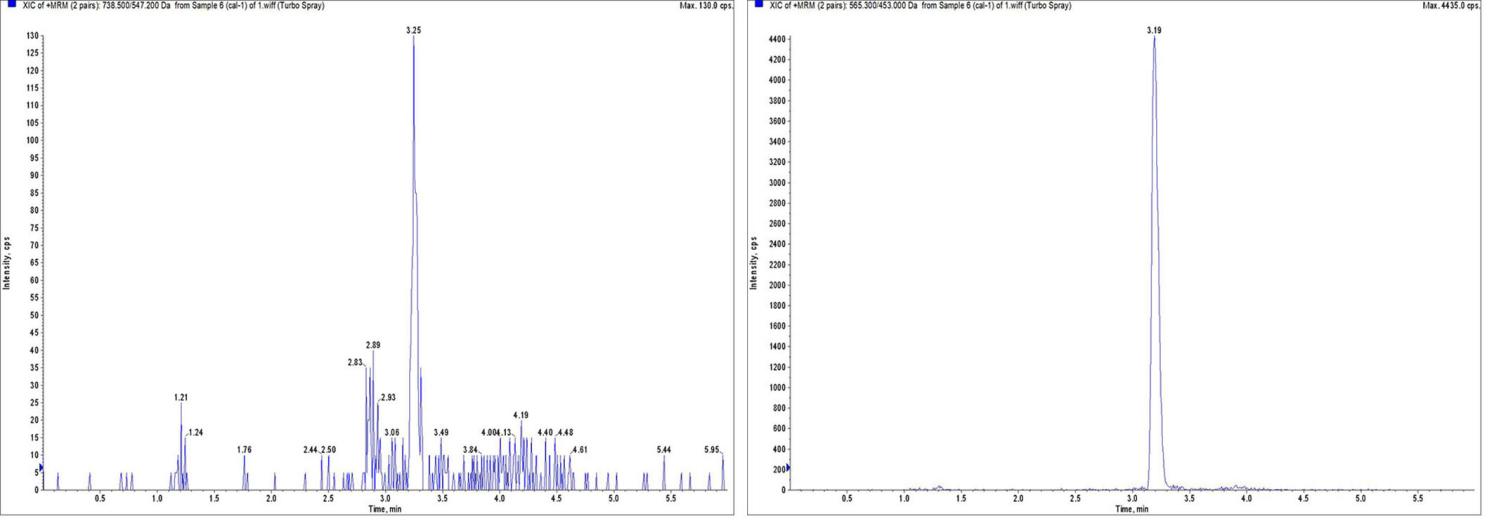


**Supplementary Figure 2C.** Chromatogram of 0.5 ng·mL^-1^ DM1 cynomolgus monkey serum sample and internal standard.


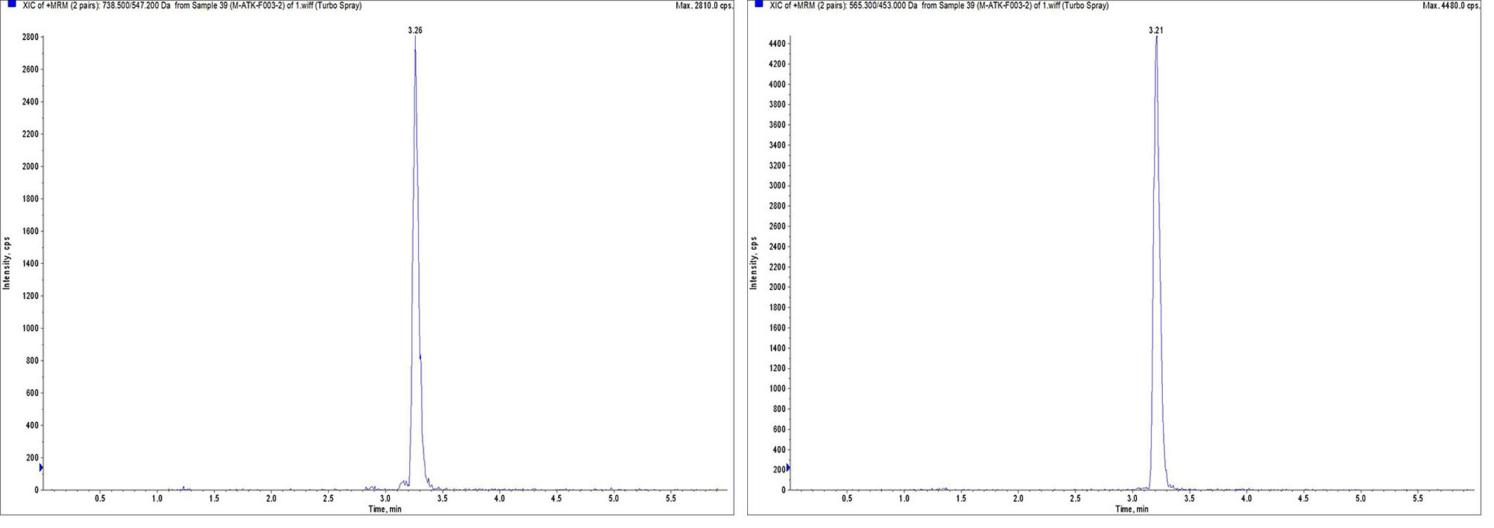


**Supplementary Figure 2D.** Chromatogram of DM1 at 0.5h cynomolgus monkeys serum samples post dose and internal standard.

## Accuracy and precision

The lower limit of quantification (LOQ) and quality control (QC) samples of DM1 at concentrations of 0.5, 1, 25, and 200 ng·mL^-1^ were spiked into cynomolgus monkey serum for test, as Supplementary Table 1 shown, the accuracy of the LOQ was within the range of 80.7% – 116%, while the accuracy of QC samples was within the range of 85.2% – 115%, respectively. The intra-assay precision ranged from 2.93% to 10.91%, and the inter-assay precision was in the range of 4.31%–10.38%.

Supplementary Table 1 Accuracy and precision results for the quantitation of DM1

| **Sample** | | **Concentration(ng·mL^-1^)** | | | | | | | |
| --- | --- | --- | --- | --- | --- | --- | --- | --- | --- |
|  |  | 0.5 | Accuracy（100%） | 1 | Accuracy（100%） | 25 | Accuracy（100%） | 200 | Accuracy（100%） |
| **1st** | 1 | / |  | 1.11 | 111 | 25.3 | 101 | 224 | 112 |
|  | 2 | 0.461 | 92.2 | 0.864 | 86.4 | 22.5 | 90 | 230 | 115 |
|  | 3 | 0.513 | 103 | 1.05 | 105 | 23.2 | 93 | 217 | 108 |
|  | 4 | 0.412 | 82.4 | 1.08 | 108 | 23.3 | 93.1 | 198 | 99.2 |
|  | 5 | 0.547 | 109 | 0.996 | 99.6 | / |  | 230 | 115 |
|  | 6 | 0.461 | 92.2 | 1.01 | 101 | 22.5 | 90 | 224 | 112 |
|  | SD | 0.05 |  | 0.09 |  | 1.15 |  | 12.03 |  |
|  | Mean | 0.48 |  | 1.02 |  | 23.36 |  | 220.50 |  |
|  | Rsd | 10.91 |  | 8.52 |  | 4.91 |  | 5.46 |  |
| **2ed** | 1 | 0.58 | 116 | 1.11 | 111 | 22.9 | 91.8 | 192 | 96 |
|  | 2 | 0.484 | 96.7 | 1.08 | 108 | 23.7 | 94.7 | 184 | 91.8 |
|  | 3 | 0.442 | 88.4 | 1.06 | 106 | 23.8 | 95.2 | 203 | 102 |
|  | 4 | 0.479 | 95.8 | 0.97 | 97 | 24.8 | 99.1 | 174 | 87 |
|  | 5 | 0.472 | 94.4 | 0.9 | 90 | 23.2 | 93 | 170 | 85.2 |
|  | 6 | 0.497 | 99.4 | 1.08 | 108 | 24.3 | 97.4 | 183 | 91.4 |
|  | SD | 0.05 |  | 0.08 |  | 0.70 |  | 12.01 |  |
|  | Mean | 0.49 |  | 1.03 |  | 23.78 |  | 184.33 |  |
|  | Rsd | 9.48 |  | 7.83 |  | 2.93 |  | 6.52 |  |
| **3rd** | 1 | 0.472 | 94.4 | 0.922 | 92.2 | 23.3 | 93.3 | 202 | 101 |
|  | 2 | 0.484 | 96.8 | 0.878 | 87.8 | 21.8 | 87.2 | 193 | 96.6 |
|  | 3 | 0.454 | 90.8 | 1.08 | 108 | 21.8 | 87.2 | 201 | 100 |
|  | 4 | 0.506 | 101 | 1.15 | 115 | 22 | 88.1 | 196 | 98 |
|  | 5 | 0.442 | 88.5 | 1.02 | 102 | 24 | 95.9 | 170 | 85.2 |
|  | 6 | 0.403 | 80.7 | 1.13 | 113 | 23.9 | 95.6 | 171 | 85.4 |
|  | SD | 0.04 |  | 0.11 |  | 1.05 |  | 14.58 |  |
|  | Mean | 0.46 |  | 1.03 |  | 22.80 |  | 188.83 |  |
|  | Rsd | 7.80 |  | 10.79 |  | 4.62 |  | 7.72 |  |
| **Inter-assay** | SD | 0.04 |  | 0.09 |  | 1.00 |  | 20.54 |  |
|  | Mean | 0.48 |  | 1.03 |  | 23.31 |  | 197.89 |  |
|  | Rsd | 9.29 |  | 8.61 |  | 4.31 |  | 10.38 |  |

Note: "" / "" the data was abnormal.

## Lower limit of quantification (LOQ)

Under the conditions where the aforementioned precision and accuracy criteria are met, the lower limit of quantification (LOQ) of the present method is 0.5 ng·mL^-1^.

Supplementary Table 2 Standard curve and linearity range

| **Theoretical concentration (ng·mL^-1^)** | **Found concentration (ng·mL^-1^)** | **MEAN±SD** | **CV%** |
| --- | --- | --- | --- |
| 0.5 | 0.471 | 0.471±0.04 | 8.01 |
|  | 0.52 |  |  |
|  | 0.478 |  |  |
|  | 0.473 |  |  |
|  | 0.414 |  |  |

## Stability

The stability of DM1 remained unaffected under the following conditions: the supernatant of processed cynomolgus monkey serum was stored in the autosampler at 4℃ for 12 hours; the drug-containing serum was stored at 4℃ for 2 hours; the drug-containing serum underwent three freeze-thaw cycles; and the drug-containing serum was stored at -80℃ for 24 months.

Supplementary Table 3 QC sample stability results (n = 4)

| **Process condition** | **Supernatant**  **4℃ 12h** | | **Serum 4℃2h** | | **Freeze-thraw 3 cycles** | | **Freeze-80℃ 24month** | |
| --- | --- | --- | --- | --- | --- | --- | --- | --- |
| **Expected concentration (ng·mL^-1^)** | 1 | 200 | 1 | 200 | 1 | 200 | 1 | 200 |
| **Obeserved concentration**  **(ng·mL^-1^)** | 0.923 | 191 | - | 208 | 1.6 | 206 | - | 197 |
|  | 0.919 | 178 | 0.867 | 218 | 0.974 | 207 | 1.12 | 184 |
|  | 0.959 | 186 | 1.03 | 218 | 1.09 | 217 | 1.11 | 176 |
|  | 1.01 | 187 | 0.85 | 223 | 1.07 | 231 | 1.15 | 192 |
| **SD** | 0.04 | 5.45 | 0.10 | 6.29 | 0.28 | 11.62 | 0.02 | 9.22 |
| **Mean** | 0.95 | 185.50 | 0.92 | 216.75 | 1.18 | 215.25 | 1.13 | 187.25 |
| **Rsd** | 4.43 | 2.94 | 10.85 | 2.90 | 23.85 | 5.40 | 1.85 | 4.92 |

## Matrix effect

By investigating the matrix effect, the coefficients of variation (CV) for the internal standard-normalized matrix factors at 1 and 200 ng·mL^-1^ concentrations were calculated to be 8.67% and 11.56%, respectively.

Supplementary Table 4 Results of Matrix Effect for DM1 in Cynomolgus Monkey Serum (n=4)

|  |  | **DM1 (ng·mL^-1^)** | | | | **IS (ng·mL^-1^)** | | | | **Internal standard normalized matrix factor** | |
| --- | --- | --- | --- | --- | --- | --- | --- | --- | --- | --- | --- |
|  |  | 1 | At/A_0_ | 200 | At/A_0_ | 1 | At/A_0_ | 200 | At/A_0_ | 1 (DM1 _At/A0_/IS_At/A0_) | 200 (DM1 _At/A0_/IS_At/A0_) |
| **A0** | | 1630 |  | 327000 |  | 31200 |  | 30200 |  |  |  |
|  |  | 1660 |  | 303000 |  | 30300 |  | 27900 |  |  |  |
|  |  | 1590 |  | 308000 |  | 28800 |  | 28800 |  |  |  |
|  |  | 1980 |  | 302000 |  | 35600 |  | 28400 |  |  |  |
| **At** | **1** | 1240 | 0.761 | 242000 | 0.740 | 21600 | 0.692 | 20900 | 0.692 | 1.099 | 1.069 |
|  |  | 1320 | 0.795 | 167000 | 0.551 | 23000 | 0.759 | 19200 | 0.688 | 1.048 | 0.801 |
|  |  | 1330 | 0.836 | 162000 | 0.526 | 21500 | 0.747 | 18300 | 0.635 | 1.120 | 0.828 |
|  |  | 1300 | 0.657 | 154000 | 0.510 | 20100 | 0.565 | 18000 | 0.634 | 1.163 | 0.805 |
|  | 2 | 1240 | 0.761 | 225000 | 0.688 | 19100 | 0.612 | 21600 | 0.715 | 1.243 | 0.962 |
|  |  | 1250 | 0.753 | 164000 | 0.541 | 19900 | 0.657 | 19900 | 0.713 | 1.147 | 0.759 |
|  |  | 1230 | 0.774 | 150000 | 0.487 | 20100 | 0.698 | 17300 | 0.601 | 1.108 | 0.811 |
|  |  | 1200 | 0.606 | 148000 | 0.490 | 19800 | 0.556 | 17500 | 0.616 | 1.090 | 0.795 |
|  | 3 | 1010 | 0.620 | 236000 | 0.722 | 18600 | 0.596 | 22800 | 0.755 | 1.039 | 0.956 |
|  |  | 1110 | 0.669 | 189000 | 0.624 | 19900 | 0.657 | 23300 | 0.835 | 1.018 | 0.747 |
|  |  | 1330 | 0.836 | 153000 | 0.497 | 20900 | 0.726 | 18600 | 0.646 | 1.153 | 0.769 |
|  |  | 1090 | 0.551 | 152000 | 0.503 | 20000 | 0.562 | 17200 | 0.606 | 0.980 | 0.831 |
|  | 4 | 1240 | 0.761 | 207000 | 0.633 | 19900 | 0.638 | 19500 | 0.646 | 1.193 | 0.980 |
|  |  | 1350 | 0.813 | 146000 | 0.482 | 20600 | 0.680 | 18100 | 0.649 | 1.196 | 0.743 |
|  |  | 1250 | 0.786 | 131000 | 0.425 | 21300 | 0.740 | 15600 | 0.542 | 1.063 | 0.785 |
|  |  | 1200 | 0.606 | 131000 | 0.434 | 21000 | 0.590 | 15100 | 0.532 | 1.027 | 0.816 |
|  | 5 | 1040 | 0.638 | 194000 | 0.593 | 19300 | 0.619 | 18400 | 0.609 | 1.031 | 0.974 |
|  |  | 1010 | 0.608 | 223000 | 0.736 | 22000 | 0.726 | 22000 | 0.789 | 0.838 | 0.933 |
|  |  | 1210 | 0.761 | 191000 | 0.620 | 21900 | 0.760 | 19000 | 0.660 | 1.001 | 0.940 |
|  |  | 1080 | 0.663 | 191000 | 0.632 | 20500 | 0.576 | 19000 | 0.669 | 1.151 | 0.945 |
|  | 6 | 921 | 0.565 | 224000 | 0.685 | 18600 | 0.596 | 21400 | 0.709 | 0.948 | 0.967 |
|  |  | 1120 | 0.675 | 164000 | 0.541 | 21500 | 0.710 | 19600 | 0.703 | 0.951 | 0.770 |
|  |  | 1090 | 0.686 | 165000 | 0.536 | 17800 | 0.618 | 20300 | 0.705 | 1.109 | 0.760 |
|  |  | 1130 | 0.571 | 170000 | 0.563 | 18100 | 0.508 | 22000 | 0.775 | 1.122 | 0.727 |
| **SD** | |  |  |  |  |  |  |  |  | 0.093 | 0.099 |
| **Mean** | |  |  |  |  |  |  |  |  | 1.077 | 0.853 |
| **Rsd** | |  |  |  |  |  |  |  |  | 8.67 | 11.56 |

## Summary

The method validation results demonstrated that this method is suitable for the determination of DM1 concentration in cynomolgus monkey serum. The specificity, sensitivity, linear range, precision, and accuracy comply with the requirements for non-clinical pharmacokinetic studies.
