# Supplementary material for: Nonclinical evaluation of HS630, a proposed biosimilar of trastuzumab emtansine: affinity, pharmacokinetics, and immunogenicity
Source: Front Pharmacol. 2025 Dec 18;16:1698727. doi: 10.3389/fphar.2025.1698727 (PMC12756435; doi:10.3389/fphar.2025.1698727)
Supplement: Supplementary file 3 [file Supplementaryfile4.doc]

Appendix IIII Concentration of total antibody and ADC after tail vein injection of HS630 and Kadcyla^®^ in tumor-bearing mice

Supplementary Table 1 Concentration of total antibody (including naked antibody and ADC) in serum after tail vein injection of 10mg·kg^-1^ HS630 in tumour bearing mice

| **Time** | **5min** | **4h** | **24h** | **48h** | **72h** | **96h** | **168h** |
| --- | --- | --- | --- | --- | --- | --- | --- |
| **Concentration (µg·mL^-1^)** | 280.042 | 165.381 | 142.168 | 101.432 | 65.309 | 50.939 | 5.206 |
|  | 294.447 | 170.116 | 145.617 | 91.462 | 64.390 | 52.042 | 6.881 |
|  | 301.624 | 162.425 | 151.604 | 91.696 | 63.554 | 55.082 | 5.004 |
| **mean** | 292.038 | 165.974 | 146.463 | 94.863 | 64.418 | 52.687 | 5.697 |
| **std** | 10.991 | 3.879 | 4.775 | 5.690 | 0.878 | 2.146 | 1.030 |

Supplementary Table 2 Concentration of total antibody (including naked antibody and ADC) in serum after tail vein injection of 10mg·kg^-1^ Kadcyla^®^ in tumour bearing mice

| **Time** | **5min** | **4h** | **24h** | **48h** | **72h** | **96h** | **168h** |
| --- | --- | --- | --- | --- | --- | --- | --- |
| **Concentration (µg·mL^-1^)** | 313.931 | 191.586 | 173.557 | 100.645 | 67.969 | 61.731 | 5.562 |
|  | 305.493 | 202.316 | 169.181 | 103.808 | 72.520 | 59.550 | 5.699 |
|  | 303.132 | 207.369 | 159.174 | 107.738 | 71.389 | 61.190 | 6.051 |
| **mean** | 307.519 | 200.424 | 167.304 | 104.064 | 70.626 | 60.824 | 5.771 |
| **std** | 5.677 | 8.060 | 7.373 | 3.553 | 2.369 | 1.136 | 0.252 |

Supplementary Table 3 Comparison of total antibody concentration (including naked antibody and ADC) in serum after tail vein injection of 10mg·kg^-1^ HS630 and Kadcyla^®^ in tumour bearing mice (µg·mL^-1^)

| **Time** | **HS630** | **Kadcyla^®^** |
| --- | --- | --- |
| 5min | 292.038±10.991 | 307.519±5.677 |
| 4h | 165.974±3.879 | 200.424±8.060 |
| 24h | 146.463±4.775 | 167.304±7.373 |
| 48h | 94.863±5.690 | 104.064±3.553 |
| 72h | 64.418±0.878 | 70.626±2.369 |
| 96h | 52.687±2.146 | 60.824±1.136 |
| 168h | 5.697±1.030 | 5.771±0.252 |

Supplementary Table 4 Concentration of ADC in serum after tail vein injection of 10mg·kg^-1^ HS630 in tumour bearing mice

| **Time** | **5min** | **4h** | **24h** | **48h** | **72h** | **96h** | **168h** |
| --- | --- | --- | --- | --- | --- | --- | --- |
| **Concentration (µg·mL^-1^)** | 141.854 | 102.119 | 80.911 | 67.364 | 25.739 | 18.918 | 2.662 |
|  | 162.062 | 92.567 | 83.579 | 65.889 | 27.893 | 19.493 | 2.371 |
|  | 145.970 | 98.066 | 84.387 | 56.131 | 28.834 | 22.398 | 2.208 |
| **mean** | 149.962 | 97.584 | 82.959 | 63.128 | 27.489 | 20.270 | 2.414 |
| **std** | 10.679 | 4.794 | 1.819 | 6.104 | 1.587 | 1.865 | 0.230 |

Supplementary Table 5 Concentration of total antibody ADC in serum after tail vein injection of 10mg·kg^-1^ Kadcyla^®^ in tumour bearing mice

| **Time** | **5min** | **4h** | **24h** | **48h** | **72h** | **96h** | **168h** |
| --- | --- | --- | --- | --- | --- | --- | --- |
| **Concentration (µg·mL^-1^)** | 158.904 | 123.377 | 116.502 | 74.107 | 32.802 | 22.825 | 3.294 |
|  | 153.109 | 138.340 | 111.496 | 77.413 | 36.917 | 20.747 | 3.304 |
|  | 165.111 | 149.648 | 110.805 | 76.801 | 34.097 | 21.188 | 3.454 |
| **mean** | 159.041 | 137.122 | 112.934 | 76.107 | 34.605 | 21.587 | 3.351 |
| **std** | 6.002 | 13.178 | 3.109 | 1.759 | 2.104 | 1.095 | 0.090 |

Supplementary Table 6 Comparison of ADC concentration in serum after tail vein injection of 10mg·kg^-1^ HS630 and Kadcyla^®^ in tumour bearing mice (µg·mL^-1^)

| **Time** | **HS630** | **Kadcyla^®^** |
| --- | --- | --- |
| 5min | 149.962±10.679 | 159.041±6.002 |
| 4h | 97.584±4.794 | 137.122±13.178 |
| 24h | 82.959±1.819 | 112.934±3.109 |
| 48h | 63.128±6.104 | 76.107±1.759 |
| 72h | 27.489±1.587 | 34.605±2.104 |
| 96h | 20.270±1.865 | 21.587±1.095 |
| 168h | 2.414±0.230 | 3.351±0.090 |

Supplementary Table 7 Concentration of total antibody (including naked antibody and ADC) in tumor after tail vein injection of 10mg·kg^-1^ HS630 in tumour bearing mice

| **Time** | **5min** | **4h** | **24h** | **48h** | **72h** | **96h** | **168h** |
| --- | --- | --- | --- | --- | --- | --- | --- |
| **Concentration (ng·g^-1^)** | 175.670 | 442.400 | 1297.040 | 778.480 | 720.760 | 454.640 | 186.610 |
|  | 169.010 | 502.460 | 1192.740 | 934.680 | 628.590 | 436.360 | 156.910 |
|  | 168.900 | 434.220 | 1019.960 | 856.660 | 642.910 | 774.910 | 188.450 |
| **mean** | 171.193 | 459.693 | 1169.913 | 856.607 | 664.087 | 555.303 | 177.323 |
| **std** | 3.877 | 37.262 | 139.943 | 78.100 | 49.600 | 190.404 | 17.702 |

Supplementary Table 8 Concentration of total antibody (including naked antibody and ADC) in serum after tail vein injection of 10mg·kg^-1^ Kadcyla^®^ in tumour bearing mice

| **Time** | **5min** | **4h** | **24h** | **48h** | **72h** | **96h** | **168h** |
| --- | --- | --- | --- | --- | --- | --- | --- |
| **Concentration (ng·g^-1^)** | 355.280 | 538.100 | 1013.280 | 987.700 | 568.140 | 488.410 | 203.580 |
|  | 149.090 | 895.130 | 1349.780 | 956.100 | 777.070 | 404.230 | 125.520 |
|  | 100.180 | 911.880 | 1198.150 | 607.690 | 425.930 | 529.040 | 262.120 |
| **mean** | 201.517 | 781.703 | 1187.070 | 850.497 | 590.380 | 473.893 | 197.073 |
| **std** | 135.390 | 211.133 | 168.523 | 210.870 | 176.623 | 63.659 | 68.532 |

Supplementary Table 9 Comparison of total antibody concentration (including naked antibody and ADC) in tumor after tail vein injection of 10mg·kg^-1^ HS630 and Kadcyla^®^ in tumour bearing mice (ng·g^-1^)

| **Time** | **HS630** | **Kadcyla^®^** |
| --- | --- | --- |
| 5min | 171.193±3.877 | 201.517±135.390 |
| 4h | 459.693±37.262 | 781.703±211.133 |
| 24h | 1169.913±139.943 | 1187.070±168.523 |
| 48h | 856.607±78.100 | 850.497±210.870 |
| 72h | 664.087±49.600 | 590.380±176.623 |
| 96h | 555.303±190.404 | 473.893±63.659 |
| 168h | 177.323±17.702 | 197.073±68.532 |

Supplementary Table 10 Concentration of ADC in tomor after tail vein injection of 10mg·kg^-1^ HS630 in tumour bearing mice

| **Time** | **5min** | **4h** | **24h** | **48h** | **72h** | **96h** | **168h** |
| --- | --- | --- | --- | --- | --- | --- | --- |
| **Concentration (µg·mL^-1^)** | 2.321 | ND | ND | ND | 1.632 | ND | 1.469 |
|  | 1.686 | ND | ND | 3.172 | 2.447 | ND | 2.010 |
|  | 2.664 | ND | 4.033 | 1.779 | ND | ND | 1.240 |

Supplementary Table 11 Concentration of total antibody ADC in tumor after tail vein injection of 10mg·kg^-1^ Kadcyla^®^ in tumour bearing mice

| **Time** | **5min** | **4h** | **24h** | **48h** | **72h** | **96h** | **168h** |
| --- | --- | --- | --- | --- | --- | --- | --- |
| **Concentration (µg·mL^-1^)** | 26.331 | 2.391 | 3.120 | 2.789 | 9.314 | 2.413 | 3.184 |
|  | 2.052 | 4.322 | 1.813 | 3.502 | 3.487 | 3.064 | 3.725 |
|  | ND | 3.136 | 2.056 | 3.040 | 3.718 | 3.826 | 4.721 |
