# Supplementary material for: Nonclinical evaluation of HS630, a proposed biosimilar of trastuzumab emtansine: affinity, pharmacokinetics, and immunogenicity
Source: Front Pharmacol. 2025 Dec 18;16:1698727. doi: 10.3389/fphar.2025.1698727 (PMC12756435; doi:10.3389/fphar.2025.1698727)
Supplement: Supplementary file 4 [file Supplementaryfile2.docx]

Appendix II Establishment and validation of the ELISA method

# Materials and Reagents

Trastuzumab (Herceptin, total antibody), manufactured by Roche was provided by Zhejiang Hisun Pharmaceutical Co., Ltd. lot number: H4159, specification: 150 mg, powder, stored at 2~8 ℃; Recomblnant human ErbB2, produced by Sino biological Inc., cat: 100040-H08H, lot: LC08SE2313. Goat anti-Human IgG-heavy and light chain monkey-adsorbed Antibody HRP Conjugated (Bethyl Laboratories, Inc.,), Skim milk (BD Difco^TM^), BSA (F. Hoffmann-La Roche&Co. Ltd), TMB single component color development solution (Beijing Solarbio Science & Technology Co.,Ltd.), H_2_SO_4_ (Sinopharm Chemical Reagent Co., Ltd.), NaCl (Xilong Chemical Reagent Co., Ltd.), KCl (Sinopharm Chemical Reagent Co., Ltd.), KH_2_PO_4_ (Sinopharm Chemical Reagent Co., Ltd.), Na_2_HPO_4_ (Sinopharm Chemical Reagent Co., Ltd.), Tween 20 (Sinopharm Chemical Reagent Co., Ltd.).

# Method validation and results

## Standard curve and linear range

Total antibody were diluted into standards of 100, 50, 25, 12.5, 6.25, 3.125, and 1.5625 ng·mL-1 with 20% monkey serum, 100 µL of each standard was taken and detected with the assay described above. The OD 450 nm-560 nm was used as the ordinate, the concentration of each standard was used as the abscissa, which was subjected to non linear regression operation, and the obtained regression equation was the standard curve of total antibody. The fitted curves are presented in Supplementary Supplementary Table 1 and Supplementary Figure 1. Calibration curves were fitted by a four parameter logistic function model, and the slope, EC50, and near linear range between the upper and lower asymptotes were evaluated. Its mathematical model is:

$$\boldsymbol{Y}\boldsymbol{=}\frac{\boldsymbol{(A}\boldsymbol{1-A}\boldsymbol{2)}}{\boldsymbol{1+}{\boldsymbol{(X}\boldsymbol{/}\boldsymbol{X}\boldsymbol{0)}}^{\boldsymbol{p}}}\boldsymbol{+}\boldsymbol{A}\boldsymbol{2}$$

Where A1 is the estimate of the lower end asymptotes of the sigmoidal curve, and A2 is the estimate of the upper end asymptotes of the sigmoidal curve. For samples with content outside the near linear range between the upper and lower asymptotes, dilute appropriately with diluent to the calibration curve optimal measured concentration range for the assay. Unknown serum samples concentration were calculated with their respective standard calibration curves on the same plate.

Supplementary Table 1 Standard curve and linearity range

| **Expected (ng·mL^-1^)** | **OD1** | **OD2** | **Mean** | **Mean-KB** | **SD** |
| --- | --- | --- | --- | --- | --- |
| 1.5625 | 0.0528 | 0.0499 | 0.0514 | 0.028 | 0.0021 |
| 3.125 | 0.0978 | 0.0940 | 0.0959 | 0.073 | 0.0027 |
| 6.25 | 0.1677 | 0.1606 | 0.1642 | 0.141 | 0.0050 |
| 12.5 | 0.3184 | 0.3132 | 0.3158 | 0.293 | 0.0037 |
| 25 | 0.6168 | 0.5719 | 0.5944 | 0.571 | 0.0317 |
| 50 | 1.0639 | 1.0390 | 1.0515 | 1.029 | 0.0176 |
| 100 | 1.7519 | 1.6817 | 1.7168 | 1.694 | 0.0496 |
| KB | 0.0161 | 0.0297 | 0.0229 | 0.000 | 0.0096 |

Note: KB for blank wells.


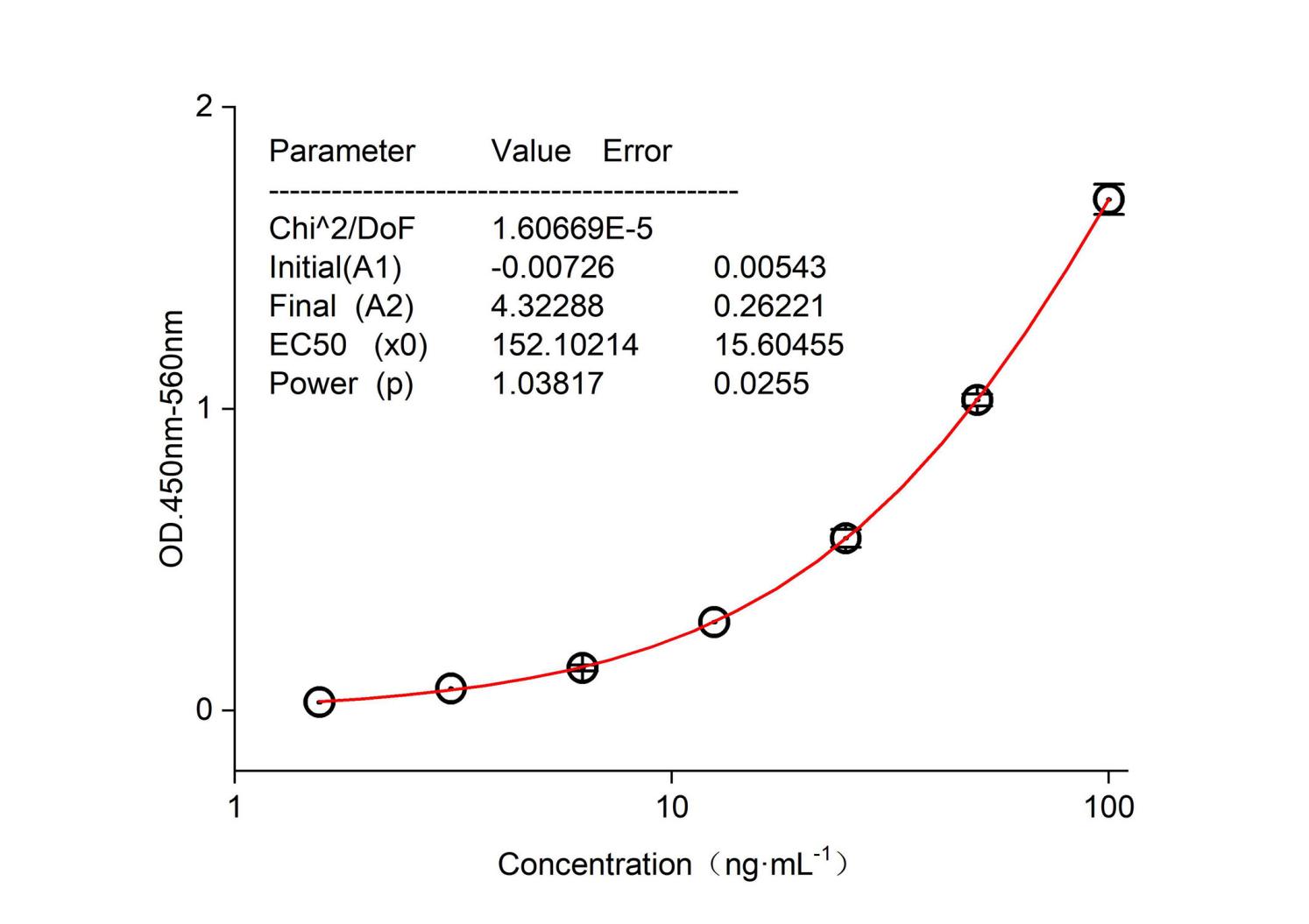


**Supplementary Figure 1.** Four-Parameter Logistic calibration curve of total antibody

## Sensitivity and the lowest concentration of detection (LOQ)

Total antibody Trastuzumab at a concentration of 1.5625 ng·mL^-1^ was made according to the method for preparing the standard curve, the assay was performed similarly as described above, repeated 8 times, and the concentration calculated from the standard curve regression equation on the same plate was the detected amount of total antibody. The standard recovery test met the LOQ of 1.5625 ng·mL^-1^ with intra assay CV% < 20% (4.87%).

Supplementary Table 2 Sensitivity of total antibody by ELISA methods with spiked in cynomolgus monkey serum

| **Expected (ng·mL^-1^)** | **Measured values**  **(ng·mL^-1^)** | **RE(%)** | **Mean RE(%)** | **CV(%)** |
| --- | --- | --- | --- | --- |
| 1.5625 | 1.9809 | +26.78 | +14.58 | 4.87 |
|  | 1.7807 | +13.96 |  |  |
|  | 1.8379 | +17.63 |  |  |
|  | 1.7766 | +13.70 |  |  |
|  | 1.6907 | +8.20 |  |  |
|  | 1.7602 | +12.65 |  |  |
|  | 1.7439 | +11.61 |  |  |
|  | 1.7521 | +12.13 |  |  |

## Precision and accuracy

The assay was performed similarly as described above for measured high, medium and low concentrations of 80, 16, 2 ng·mL^-1^, and each concentration was assayed 8 times in parallel for a total of 3 times to calculate the intra assay CV, inter assay CV values and the RE values, respectively. The results showed that the intra assay and inter assay CVS at the three concentration levels, high, medium and low, were less than 15%, indicated the detection method showed high precision; At the same time, the RE values between the intra - and inter assay at the high, medium and low concentration levels were less than 15%, indicated the detection method showed high accuracy.

Supplementary Table 3 Precision and accuracy detection of HS630 by ELISA methods with spiked in cynomolgus monkey serum

|  | **Expected (ng·mL^-1^)** | **Measured (ng·mL^-1^)** | **CV%** | **RE%** |
| --- | --- | --- | --- | --- |
| **Intra assay（n=8）** | 2 | 2.00±0.19 | 9.60 | +0.16 |
|  | 16 | 16.31±1.06 | 6.51 | +1.93 |
|  | 80 | 75.06±4.47 | 5.96 | -6.18 |
| **Inter assay（n=24）** | 2 | 1.97±0.19 | 9.43 | -1.28 |
|  | 16 | 16.30±1.00 | 6.17 | +1.65 |
|  | 80 | 74.97±4.28 | 5.71 | -6.32 |

## Specificity

Trastuzumab was prepared to 100 ng·mL^-1^-1.5625 ng·mL^-1^ as the method for preparing the standard curve described above, while.the same concentration gradient samples were prepared for HS630, Kadcyla^®^, Bevacizumab, Adalimumab and Infliximab, and the detection results are shown in Supplementary Figure 2, Trastuzumab showed no cross reactivity with Bevacizumab, Adalimumab and Infliximab, and good methodological specificity.There were cross reactivity among Herceptin, HS630, and Kadcyla^®^, indicated that the detection method was capable of simultaneously detecting free Herceptin, HS630, and Kadcyla^®^ in test samples. Therefore, this method was called a total antibody detection method.


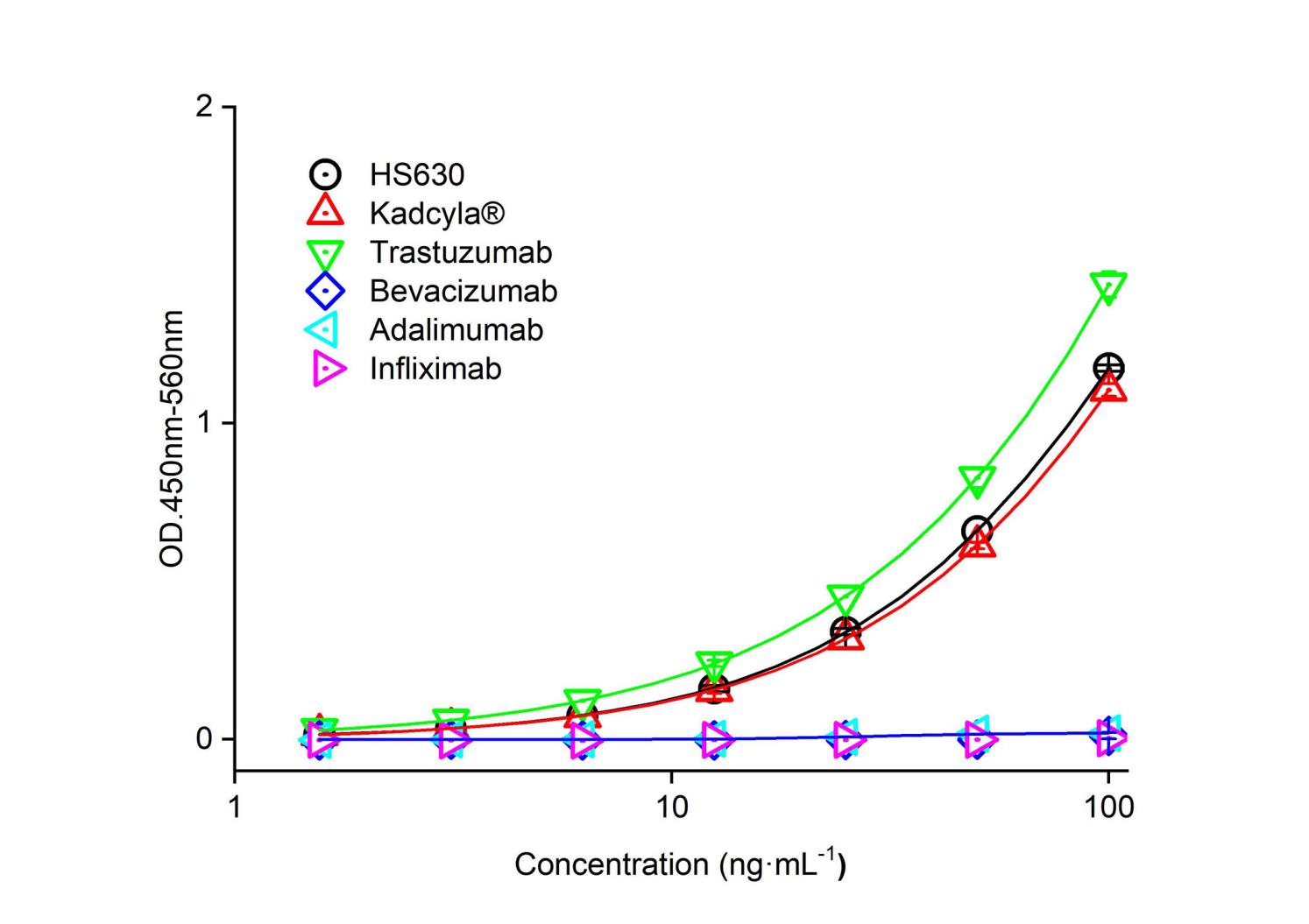


**Supplementary Figure 2.** Specificity validation curve of total antibody

## Stability

The results of the stability study of total antibody in monkey serum are presented in Supplementary Table 4, and the results indicate that the total antibody in serum stored at -80 ℃ (after three freeze/thaw cycles), at room temperature for 4 h, at 4 ℃ for 4 h, at -80 ℃ for 40 days, at 100 days and at 160 days does not affect its stability. However, considered the long-term storage at -80 ℃ for 160 days of HS630 did not guarantee its stability, therefore the sample detection were all completed before 160 days.

Supplementary Table 4-1 Stability of total antibody (unit: ng·mL^-1^)

| **Treatment conditions** | **Freeze thawed 3 times** | | |  | **Room temperature 4 h** | | |  | **4℃ 4h** | | |  |
| --- | --- | --- | --- | --- | --- | --- | --- | --- | --- | --- | --- | --- |
| **Expected** | 2 | 16 | 80 |  | 2 | 16 | 80 |  | 2 | 16 | 80 |  |
| **Measured values** | 2.28 | 17.18 | 82.84 |  | 2.33 | 17.22 | 82.21 |  | 2.33 | 17.20 | 82.43 |  |
|  | 1.82 | 15.86 | 76.23 |  | 1.86 | 15.88 | 76.87 |  | 1.86 | 15.88 | 76.55 |  |
|  | 1.78 | 15.30 | 70.57 |  | 1.85 | 15.31 | 70.27 |  | 1.84 | 15.29 | 70.64 |  |
|  | 1.73 | 15.23 | 67.88 |  | 1.79 | 15.25 | 69.18 |  | 1.79 | 15.27 | 68.55 |  |
|  | 1.90 | 15.43 | 74.42 |  | 1.95 | 15.43 | 72.72 |  | 1.95 | 15.44 | 73.61 |  |
|  | 1.85 | 16.14 | 72.62 |  | 1.89 | 16.17 | 72.71 |  | 1.86 | 16.14 | 72.58 |  |
|  | 2.16 | 16.86 | 78.30 |  | 2.19 | 16.86 | 76.09 |  | 2.18 | 16.85 | 77.29 |  |
|  | 2.11 | 18.09 | 82.57 |  | 2.15 | 18.20 | 80.11 |  | 2.14 | 18.16 | 81.29 |  |
| **Mean** | 1.96 | 16.26 | 75.68 |  | 2.00 | 16.29 | 75.02 |  | 1.99 | 16.28 | 75.37 |  |
| **RE(%)** | -2.21 | +1.63 | -5.40 |  | +0.08 | +1.82 | -6.22 |  | -0.34 | +1.75 | -5.79 |  |
| **SD** | 0.20 | 1.03 | 5.40 |  | 0.19 | 1.05 | 4.62 |  | 0.20 | 1.04 | 4.92 |  |
| **CV(%)** | 10.29 | 6.32 | 7.13 |  | 9.69 | 6.47 | 6.16 |  | 10.01 | 6.40 | 6.53 |  |

Supplementary Table 4-2 Stability of total antibody (unit: ng·mL^-1^)

| **Treatment conditions** | **Stored at-80 °C for 40 days** | | |  | **Stored at-80 °C for 100 days** | | |  | **Stored at-80 °C for 160 days** | | |  |
| --- | --- | --- | --- | --- | --- | --- | --- | --- | --- | --- | --- | --- |
| **Expected** | 2 | 16 | 80 |  | 2 | 16 | 80 |  | 2 | 16 | 80 |  |
| **Measured values** | 2.33 | 17.14 | 83.35 |  | 2.25 | 16.90 | 89.32 |  | 2.26 | 17.08 | 86.46 |  |
|  | 1.80 | 15.83 | 74.77 |  | 1.70 | 15.73 | 76.34 |  | 1.74 | 15.82 | 75.99 |  |
|  | 1.79 | 15.36 | 68.97 |  | 1.68 | 15.19 | 69.35 |  | 1.71 | 15.23 | 69.53 |  |
|  | 1.73 | 15.28 | 65.37 |  | 1.63 | 15.15 | 64.43 |  | 1.67 | 15.21 | 65.23 |  |
|  | 1.94 | 15.43 | 76.15 |  | 1.83 | 15.35 | 80.75 |  | 1.86 | 15.38 | 78.48 |  |
|  | 1.96 | 16.16 | 72.22 |  | 1.88 | 15.65 | 74.04 |  | 1.79 | 16.12 | 73.46 |  |
|  | 2.17 | 16.83 | 81.43 |  | 2.10 | 16.72 | 89.31 |  | 2.10 | 16.76 | 84.91 |  |
|  | 2.14 | 17.87 | 85.14 |  | 2.07 | 18.00 | 99.97 |  | 2.06 | 18.05 | 92.61 |  |
| **Mean** | 1.98 | 16.24 | 75.93 |  | 1.89 | 16.09 | 80.44 |  | 1.90 | 16.20 | 78.34 |  |
| **RE(%)** | -0.89 | +1.48 | -5.09 |  | -5.34 | +0.54 | 0.55 |  | -5.11 | +1.28 | -2.08 |  |
| **SD** | 0.21 | 0.95 | 7.02 |  | 0.23 | 1.02 | 11.81 |  | 0.22 | 1.02 | 9.19 |  |
| **CV(%)** | 10.61 | 5.85 | 9.25 |  | 11.93 | 6.34 | 14.68 |  | 11.33 | 6.28 | 11.74 |  |

## Effect of sample dilution factor

Total antibody was prepared with 20% monkey serum at a concentration of 1mg·mL^-1^, then diluted it in 12500 fold, 62500 fold, and 500000 fold with 8 times in parallel for detected. The results are shown in Supplementary Table 5. Indicating that diluting total antibody standard solution with 20% monkey serum at 12500 fold, 62500 fold, and 500000 fold had no effect on sample detection and there is no dilution effect.

Supplementary Table 5 Effect of sample dilution factor

|  | **500000 fold dilution** | **62500 fold dilution** | **12500 fold dilution** |
| --- | --- | --- | --- |
| **Measured values**  **(ng·mL^-1^)** | 2.26 | 17.17 | 83.10 |
|  | 1.79 | 15.85 | 76.05 |
|  | 1.77 | 15.29 | 69.83 |
|  | 1.72 | 15.26 | 67.36 |
|  | 1.90 | 15.44 | 75.06 |
|  | 1.83 | 16.01 | 72.62 |
|  | 2.17 | 16.83 | 79.29 |
|  | 2.08 | 18.10 | 83.85 |
| **Mean (ng·mL^-1^)** | 1.94 | 16.24 | 75.89 |
| **RE(%)** | -3.01 | +1.52 | -5.13 |
| **SD** | 0.20 | 1.03 | 5.95 |
| **CV(%)** | 10.39 | 6.32 | 7.84 |

## Summary

The linear range of total antibody ELISA method was 1.5625-100 ng·mL^-1^, the recovery of LOQ (1.5625 ng·mL^-1^) was evaluateed as intra assay CV% < 20% (4.87%).. The intra assay precision was 9.60%, 6.51%, and 5.96% for the high, medium, and low concentrations, respectively, the intra assay accuracy was +0.16%, +1.93%, and -6.18%; The inter assay precisions were 9.43%, 6.17% and 5.71% for high, medium and low concentrations, respectively, and the inter assay accuracies were -1.28%, +1.65% and -6.32%. This method had excellent specificity, there were cross reactivity among Herceptin, HS630, and Kadcyla^®^, indicated that the detection method was capable of simultaneously detecting free Herceptin, HS630, and Kadcyla^®^ in test samples. Therefore, this method was called a total antibody detection method. Total antibody in serum stored at -80 ℃ (after three freeze/thaw cycles), at room temperature for 4 h, at 4 ℃ for 4 h, at -80 ℃ for 40 days, at 100 days and at 160 days does not affect its stability. However, considered the long-term storage at -80 ℃ for 160 days of HS630 did not guarantee its stability, therefore the sample detection were all completed before 160 days. And no effect on sample detection after 12500 fold, 62500 fold, and 500000 fold dilutions were performed. The methodological validation showed that the specificity, precision and accuracy of the ELISA method for measuring total antibody concentrations in monkey serum fulfilled the requirements of pharmacokinetic studies.
